# Epihiper—A high performance computational modeling framework to support epidemic science

**DOI:** 10.1093/pnasnexus/pgae557

**Published:** 2024-12-11

**Authors:** Jiangzhuo Chen, Stefan Hoops, Henning S Mortveit, Bryan L Lewis, Dustin Machi, Parantapa Bhattacharya, Srinivasan Venkatramanan, Mandy L Wilson, Chris L Barrett, Madhav V Marathe

**Affiliations:** Biocomplexity Institute, University of Virginia, Charlottesville, VA, USA; Biocomplexity Institute, University of Virginia, Charlottesville, VA, USA; Biocomplexity Institute, University of Virginia, Charlottesville, VA, USA; Department of Systems and Information Engineering, University of Virginia, Charlottesville, VA, USA; Biocomplexity Institute, University of Virginia, Charlottesville, VA, USA; Biocomplexity Institute, University of Virginia, Charlottesville, VA, USA; Biocomplexity Institute, University of Virginia, Charlottesville, VA, USA; Biocomplexity Institute, University of Virginia, Charlottesville, VA, USA; Biocomplexity Institute, University of Virginia, Charlottesville, VA, USA; Biocomplexity Institute, University of Virginia, Charlottesville, VA, USA; Department of Computer Science, University of Virginia, Charlottesville, VA, USA; Biocomplexity Institute, University of Virginia, Charlottesville, VA, USA; Department of Computer Science, University of Virginia, Charlottesville, VA, USA

**Keywords:** computational epidemiology, high performance computing, social-contact networks, agent-based models, programmable pharmaceutical and nonpharmaceutical interventions

## Abstract

This paper describes Epihiper, a state-of-the-art, high performance computational modeling framework for epidemic science. The Epihiper modeling framework supports custom disease models, and can simulate epidemics over dynamic, large-scale networks while supporting modulation of the epidemic evolution through a set of user-programmable interventions. The nodes and edges of the social-contact network have customizable sets of static and dynamic attributes which allow the user to specify intervention target sets at a very fine-grained level; these also permit the network to be updated in response to nonpharmaceutical interventions, such as school closures. The execution of interventions is governed by trigger conditions, which are Boolean expressions formed using any of Epihiper’s primitives (e.g. the current time, transmissibility) and user-defined sets (e.g. people with work activities). Rich expressiveness, extensibility, and high-performance computing responsiveness were central design goals to ensure that the framework could effectively target realistic scenarios at the scale and detail required to support the large computational designs needed by state and federal public health policymakers in their efforts to plan and respond in the event of epidemics. The modeling framework has been used to support the CDC Scenario Modeling Hub for COVID-19 response, and was a part of a hybrid high-performance cloud system that was nominated as a finalist for the 2021 ACM Gordon Bell Special Prize for high performance computing-based COVID-19 Research.

**Significance Statement**

The Epihiper epidemic simulation model is an advanced computational tool designed to support policymakers analyzing highly detailed what-if scenarios under a range of interventions and disease strains such as for COVID-19. Targeting teams with epidemic- and computational expertise, it supports complete specification and parameterization of highly custom disease models and interventions without any modifications of the simulation code, thereby reducing response time. The models and interventions can be examined separately from the code, making their verification easier for domain experts without a computing background. Epihiper, which is openly available under the MIT license, supported state and government agencies all throughout the COVID-19 epidemic and was used for regular contributions to the Scenario Modeling Hub effort.

## Introduction

Despite significant progress in medical and public health sciences, epidemics caused by infectious diseases continue to have a global impact. The COVID-19 pandemic served as a grim reminder of the significant social, economic, and health impacts of pandemics. Computational and mathematical models have proven useful in planning and responding to epidemics in the past (1–4). Their use has steadily increased in the last two decades as policymakers and epidemiologists seek to study a range of questions, including what-if scenarios, forecasting, diverse intervention strategies, and supporting logistics (see, e.g. the overview in (5)). The COVID-19 pandemic saw extensive development and use of computational models to support policymakers and epidemiologists (6–8). The developed models ranged from descriptive, such as static estimates of correlations within large databases, to generative, such as computing the spread of different kinds of contagions via person-to-person interactions through a large population—these include the spread of a disease, as well as (mis-)information and fear about the disease (see, e.g. (9, 10)).

### Networked epidemiology

Compartmental mass action models have been used to study epidemic dynamics and complex interventions for over a century. They have been the cornerstone of epidemic science, and have been used due to their simplicity, underlying theory, and computational tractability. An alternative approach for representing epidemic processes and interventions is to use networks to explicitly represent the underlying social contact network between individual agents (see, e.g. (11–14)). Such a representation captures the underlying heterogeneity of the contact structure, and also allows us to formally and succinctly capture individual behaviors and interventions. Network-based models have become increasingly popular over the last 25 years as policymakers seek to develop targeted policies and response strategies (15). Nevertheless, such models are computationally intensive, and thus naturally motivate the use of high performance computing (HPC) for implementing these models.

### Epihiper

**Fig. 1.**
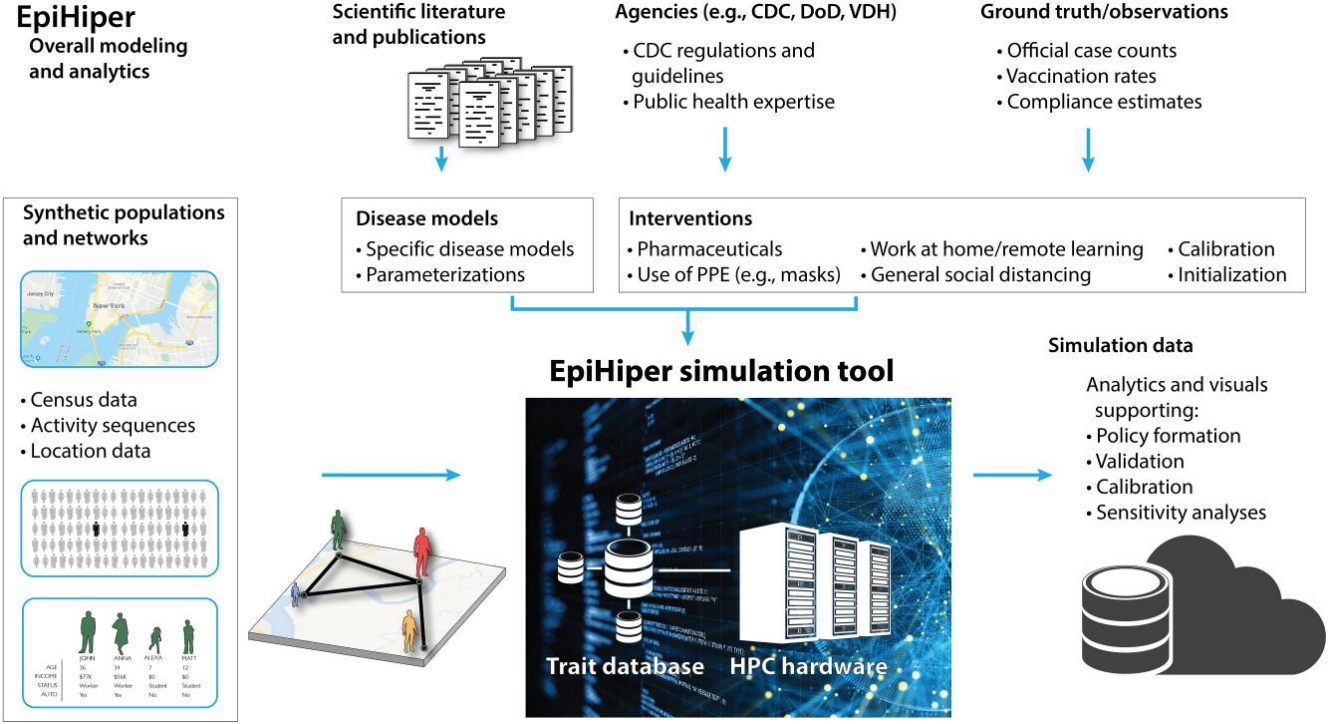
Framework overview: Epihiper takes as input a synthetic population consisting of people with individual demographic- and disease-relevant attributes, as well as a contact network on which transmission takes place (left panel). This is integrated with disease models and parameters taken from peer-reviewed literature, and highly detailed interventions based on guidelines from the Centers for Disease Control and Prevention (CDC), Department of Defense (DoD), and the Virginia Department of Health (VDH) (top panel). After calibration, the Epihiper framework can generate simulation outcomes and analytics for virtually any scenario involving COVID-19 and influenza-like diseases. For large, complex scenarios, the use of high performance computing hardware is typically needed, along with person attributes stored in a dedicated trait database (lower part of diagram; PPE, personal protective equipment).

In this article, we present Epihiper, a high performance, computational modeling framework supporting epidemic science, and which addresses many of the challenges listed in (16), including data limitations, the need for significant computing resources, and model credibility, all as discussed later in the Introduction. It has also tackled several of the open problems in network epidemiology described in (17). This framework builds on our earlier work, first published in (12) and subsequently (18–23). The design of Epihiper integrates four major components as shown in Fig. 1: (i) a labeled, time-varying social contact network over which contagions spread; (ii) fully customizable disease models capturing disease transmission between hosts, as well as within-host disease progression; (iii) user-programmable, pharmaceutical and nonpharmaceutical interventions (NPIs); and (iv) the discrete-time, parallel simulator architected to take full advantage of modern HPC hardware.

This modeling framework has been used extensively by our group throughout the ongoing COVID-19 pandemic for planning and response efforts in support of local, state, and federal authorities.

The use of Epihiper has been demonstrated over the last few years in our work with the COVID-19 Scenario Modeling Hub (SMH) (https://covid19scenariomodelinghub.org/), where it has been used for nine rounds, with each round presenting several challenges. The modeled scenarios range from limited vaccine supply (24), reduced non-pharmaceutical interventions (NPIs), emerging new variants in early 2021, vaccine hesitancy, waning immunity, and childhood vaccination in late 2021 (25). Some of this work formed the basis for one of our showcases related to HPC-based COVID-19 research, demonstrating Epihiper’s strengths and other HPC capabilities as reported in (25). Specifically, this included scaling to large networks, scaling to highly complex interventions, and accommodating large computational designs, all of which were needed in our collaborations with public health and government agencies. It has also been used in the Influenza Scenario Modeling Hub (SMH) for scenario-based projections during the 2022–2023 influenza season in the United States. Epihiper was also used to support the White House US–UK (United States–United Kingdom) challenge to develop privacy-enhancing technologies (26, 27). The target groups envisioned for Epihiper are teams with combined expertise covering epidemiology, public health, and high performance computing. In our more than decade-long experience, the work required to assist public health experts and policymakers when faced with challenges like COVID-19, or the introduction of other novel diseases, is highly nontrivial, and yet will typically have to be done in a very short time-frame. Nonetheless, we envision that tools and specialized user interfaces can be built on top of Epihiper, the latter being central in the design of Epihiper. Since the source code is made available through the MIT license, this avenue is free for anyone to pursue; the authors’ research group has already started the development of one such front-end.

### Do we need yet another modeling framework for computational epidemics?

Our group has been developing data-driven, computational modeling frameworks in support of networked epidemiology for close to two decades (12). Subsequent efforts include (18–23) by us, as well as others (28–30), and recent work related to COVID-19 planning and response, including (1, 31–39). In this body of work, smaller regions have been analyzed using detailed models, and larger regions using aggregate models. We remark that current agent-based models (ABMs) for simulating epidemic dynamics are often challenged to simultaneously scale to large networks while representing complex interventions and disease transmission models due to their software architecture. Although there are many other frameworks for computational epidemiology, many have limitations which the Epihiper project directly addresses through model design and architecture (marked (i) through (iii) in the following). One such aspect is (i) *scalability:* few existing systems scale to the network sizes considered here, which are on the order of 100 million nodes. Systems that claim scalability (but also other systems) often have limited (ii) *capabilities and expressiveness* in terms of disease modeling and interventions, a second key aspect addressed by Epihiper. Typically, disease models and/or interventions are hard-coded, making it somewhat involved for other researchers to add to or extend any of these elements, as this would require detailed knowledge of the model and its implementation. While such designs may have been the result of having to support policy decisions for COVID-19 in near real-time, it renders frameworks less flexible, frequently requiring extensions to the code base whenever a new scenario must be addressed. A framework can greatly benefit by having (iii) *external specifications of disease models and their interventions*, another key aspect addressed in Epihiper’s design. Not only does this approach support more transparent peer-review of models and interventions, it also avoids the inherent risk of introducing model errors, as well as coding errors, through frequent code changes. We argue that having these model components specified externally leads to a more flexible framework that can respond more rapidly and more reliably to the needs of public policymakers. We hope that the level of detail provided in this paper regarding the model and the mapping to its software architecture can serve as a guideline for what is expected in published work on epidemic simulation models. This is aligned with the Overview, Design concepts, and Details (ODD) protocol of (40) and seeks to ensure a basic level of reproducibility^a^ and to support validation (e.g. (42, 43).) A solution using external, text-based specification of the disease models and interventions (or their parameterizations) further helps support reproducibility. While there are other solutions, this approach has been employed with great success in the systems biology community, where they introduced the SBML (Systems Biology Markup Language) standard for exactly this use case (44). Finally, we remark that even though the focus has been on use of Epihiper to study problems in epidemic science, the framework is actually much more general. With minimal or no changes, it can be adapted to analyze a number of other phenomena such as the complex contagion models studied in (45, 46).

## Results

This section covers the features of the Epihiper epidemic model, scenarios for which it was used to inform complex decision-making in health policy, and its computational scaling capabilities. The comprehensive process and its components are illustrated in Fig. 1.

### The Epihiper disease model

The model captures epidemic spread over a social contact network of agents equipped with attributes (e.g. social, demographic, behavioral, or vaccine history). Edges represent the possibility of disease transmission and are labeled by properties such as contact duration and location type, see (25) and the Supplementary material for more details. The Supplementary material also includes an example illustrating all model components, as well as a complete example with accompanying downloadable configurations and data that the reader can execute.

**Fig. 2.**
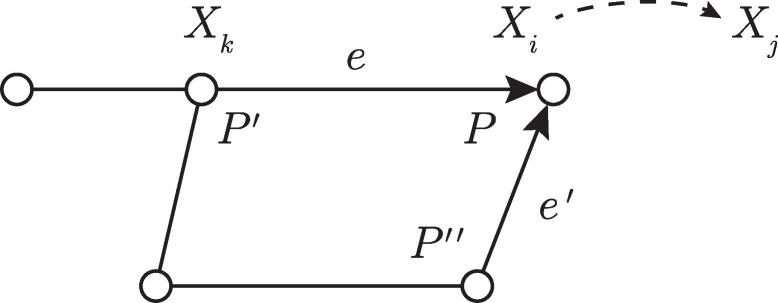
An example network for Epihiper with agents *P*, $P^{\prime }$, and $P^{\prime \prime }$.

The disease model is fully specified by the user and starts from a custom set $X=\{X_{1},X_{2},\ldots ,X_{m}\}$ of *health states*. Disease evolution is split into *disease transmission* models and within-host *disease progression* models. The disease progression model specifies the nontransmission transitions of health states $X_{i}⟶X_{j}$, the dwell-time distribution for state $X_{i}$ given that the transition $X_{i}⟶X_{j}$ takes place, and the transition probabilities $p_{i,j}$ for all successor states $X_{j}$ of $X_{i}$. For the *disease transmission model*, we refer to Fig. 2, which shows a network where a susceptible person *P* is in contact with infectious persons $P^{\prime }$ and $P^{\prime \prime }$. Focusing on the pair $\left(P^{\prime },P\right)$, we combine the state infectivity and state susceptibility of their respective health states $X_{k}$ and $X_{i}$ with the infectivity scaling factor of $P^{\prime }$ and the susceptibility scaling factor of *P* to form the propensity associated with the contact configuration $T_{i,j,k}=T(X_{i},X_{j},X_{k})$ for the potential transition of the health state of person *P* to $X_{j}$ as:


(1) $$\begin{array}{rl} \rho (P,P^{\prime },T_{i,j,k},e) & =[T\cdot \tau ]\times w_{e}\times \alpha_{e}\\ & \;\times [\beta_{\sigma }(P)\cdot \sigma (X_{i})]\times [\beta_{\iota }(P^{\prime })\cdot \iota (X_{k})]\times \omega (T_{i,j,k}) \end{array}$$

Here, *T* is the duration of contact for the edge $e=(P^{\prime },P,w_{e},\alpha_{e},T)$, $w_{e}$ is an edge weight, and $\alpha_{e}$ is a Boolean value indicating whether or not the edge is active (e.g. not disabled due to a school closure). All remaining quantities of (1) are detailed in Table 1. For each time step, and for each person *P*, the propensities *ρ* from (1) are collected across all edges *e* and contact configurations *T* as the sequence $\rho_{P}=(\rho (P,P^{\prime },T,e{)}_{P^{\prime },T,e})$. Given the sequence $\rho_{p}$, determining if *P* becomes infected is modeled as a Gillespie process (47, 48). The person $P^{\prime }$ to whom one attributes *P* becoming infected is also determined as part of this step. The full details are given in the Supplementary material. We note that the Epihiper transmission model is also referred to as a *dose–response model* (see, e.g. (49, 50)), and is structurally quite similar to the model used by MATSim-EpiSim (51, 52).

**Table 1.** Epihiper core model parameters. Here AP denotes an agent property, while DP denotes a disease property.

Parameter	Description
*P*, $P^{\prime }$	Agent/person/node
$X_{i}$	Health state *i*
$\sigma (X_{i})$	Susceptibility of health state $X_{i}$ [DP]
$\iota (X_{i})$	Infectivity of health state $X_{i}$ [DP]
$\beta_{\sigma }(P)$	Susceptibility scaling factor for person *P* [AP]
$\beta_{\iota }(P)$	Infectivity scaling factor for person *P* [AP]
$w_{e}$	Weight of edge $e=(P,P^{\prime })$
$\alpha_{e}$	Flag indicating whether the edge *e* is active
$T(X_{i},X_{j},X_{k})$	Contact configuration for a susceptible transition
	from $X_{i}$ to $X_{j}$ in the presence of state $X_{k}$ [DP]
$\omega_{i,j,k}$	Transmission weight of contact configuration
	$T(X_{i},X_{j},X_{k})$ [DP]
*τ*	Transmissibility
$\rho (P,P^{\prime },T_{i,j,k},e)$	Contact propensity

It is assumed (i) that propensities for a person are independent across contact configurations, and (ii) that, during any time step, no person can change their health state. The first assumption is quite common and not unreasonable for the contact networks that are used. The second assumption can always be accommodated by reducing the size/duration of the time step. Its real purpose is to ensure *order invariance* of contacts within a time step, thus providing the required guarantee for algorithm correctness.

In our work with the COVID-19 pandemic, the within-host models and the health states became increasingly more complex as time progressed: asymptomatic states were important during the early stages, then were followed by the need to represent vaccine uptake, which would impact, for example, the ability to transmit and become infected. Yet more states were necessary to capture immune waning and to account for multiple strains/variants and their cross-correlations.

### Epihiper interventions

Interventions are central to the Epihiper model and represent the mechanisms that allow one to capture complex pharmaceutical and nonpharmaceutical mitigation strategies. Examples include city-scale lock-downs and closures of various facilities; vaccinations, which may be temporally and spatially constrained based on production schedules; test-trace-isolate strategies; and behavioral adaptation by individual citizens, including mask wearing, vaccine hesitancy, and social distancing. It is instructive to think of Epihiper interventions as general scripts that are executed when prescribed events take place (trigger conditions), and that can modify the properties/states of the node and edge elements in the intervention’s assigned target set.

Clearly, the diversity of possible interventions is vast, which underscores the importance of having a generic and flexible approach for their representation (53). Just as for the disease model, Epihiper allows users to specify highly custom and complex interventions as an external file which is provided as part of the input configuration data. We believe that having external descriptions of disease models and interventions is one of the unique features of Epihiper. As the COVID-19 pandemic progressed, this was one of the main factors that allowed us to keep up with the continuous need for implementing increasingly complex interventions to support public policy. This was completed without modifications to the source code, thus eliminating testing and verification of such revisions and changes. Here we present an overview of Epihiper interventions. Full descriptions are provided in the Supplementary material.

An Epihiper intervention has three components: the first two are the *intervention target T* and the *trigger condition C*. The intervention target *T* is a set consisting of nodes and/or edges, and is formed using Epihiper primitives which are detailed in the Supplementary material. An example of a target is the set $T=T_{\left[5,16\right]}$ consisting of all nodes whose age *a* falls in the range 5≤a≤16, which may be used for a school closure intervention. The trigger condition *C* is a Boolean expression formed using Epihiper primitives (see the Supplementary material) and sizes of sets, e.g. $|T_{\left[5,16\right]}|\geq 25$. The final component of an Epihiper intervention is a collection of operations, the *operation ensemble*, that is applied against the states associated with the elements of the target set. The operation ensemble has three control block structures resembling those found in common programming languages, but adapted to epidemic processes and interventions: the foreach, sampling, and once. Statements within the foreach block are applied to all elements of the target *T*. The once construct is used to update variables and states not attached to any node or edge, which can be used to introduce a supply of vaccine dosages, for example. Finally, the sampling structure allows one to specify statements to be applied to a sampled subset of *T* under various sampling regimens. A person receiving a vaccine, for example, can be captured by setting that person’s susceptibility scaling factor to a suitable value depending on their attributes (e.g. age, occupation type, or health state).

All operations enter a priority queue which is sorted first by scheduled execution time, and second by priority. Within a given time step, all operations scheduled for that step are processed in sorted order. Collections of operations of equal priority are processed in random order. Finally, an operation is executed only if its associated condition is true at the time of processing. We note that one must pay careful attention when designing interventions where the order of operations matters. It is the responsibility of the person constructing the (set of) interventions to assign priorities and conditions to ensure that interventions are applied in the intended order.

### Illustrations of Epihiper capabilities

Epihiper was developed partly in response to the COVID-19 pandemic and has been used extensively to help inform analysts at the US Department of Defense (DoD) and the Defense Threat Reduction Agency (DTRA), the Virginia Department of Health (VDH), and US Centers for Disease Control and Prevention (CDC). Examples of studies performed using Epihiper include the risks of workplace outbreaks after office reopenings, impacts on neighboring communities, and evaluations examining effectiveness of contact tracing and quarantine measures. Some key findings were that contact tracing could help reduce cases, hospitalizations, and deaths, and to reach the targeted level of case numbers much faster (54). Epihiper has also been used to support the COVID-19 SMH,^b^ and the European Centre for Disease Prevention and Control.^c^ Here, modeled scenarios have included limited vaccine supply (24), reduced NPIs, emerging new variants in early 2021, vaccine hesitancy, waning immunity, and childhood vaccination in late 2021 (25). This work formed the basis for our submission (25) to the 2021 ACM Gordon Bell Prize which was selected as a finalist in the category of *HPC-Based COVID-19 Research*.

We next highlight examples showcasing specific components of Epihiper interventions followed by a more comprehensive overview of its use in SMH Round 12.

#### Disease model representation

Examples of health states represented for COVID-19 include the usual susceptible, exposed, infectious, and recovered states. In addition, we have included asymptomatic states and detailed disease outcome states such as hospitalization, ventilation, and death. Scenarios required the base disease states to be expanded by age group, and assignment of age-dependent dwell-time distributions to the states, as well as age-dependent transition probabilities between the states. Throughout the SMH rounds, we augmented the base disease model with various vaccinated states: one-dose Pfizer/Moderna, two-dose Pfizer/Moderna, Johnson & Johnson, boosters, and a partially susceptible state for nodes with waning of immunity obtained by natural infection or vaccination. We also implemented age-dependent protections against infection, transmission, and severe disease for nodes with immunity (from vaccination, infection, or waning). All of these disease model extensions were done without any change to the Epihiper source code: the only parts that were updated were the disease model input files specifying the health states and transitions between them, along with parameters that were updated to their most current values.

#### Initialization capabilities

Now that the COVID-19 pandemic has entered its fourth year, we choose to start the simulations from December 2021. Through interventions, Epihiper allows a given number of people to be sampled across age-stratified distributions at the county level, and to initialize their health states to *recovered* to account for prior infections, *vaccinated* due to prior vaccinations, to *partially susceptible* in the case of people with waned immunity, and to *infected* for active infections in December 2021. This capability allows us to start the simulation from a given snapshot with the set states for all nodes and the network. Epihiper also allows for the creation of a snapshot of nodes and network states during a simulation from which a later simulation may be resumed.

#### Intervention modeling capabilities

SMH Round 12 required representation of both NPIs and pharmaceutical interventions (PIs): (i) individual-level preventive behavior (e.g. mask wearing) with 25% compliance; (ii) individual-level social distancing (reducing daily nonessential activities) with 15% compliance; (iii) universal school closure due to winter break; and (iv) household-level voluntary home isolation of symptomatic infections with 75% compliance. For NPI (i), we changed the infectivity parameter of compliant nodes, whereas for (ii), we changed the activities of compliant nodes and removed their contacts with other people during the reduced activities. In the case of (iii), we temporarily disabled all contacts in school, and for (iv), we temporarily disabled contacts of isolated nodes with people outside their households. PIs included a collection of vaccinations that mainly moved nodes to appropriate vaccinated states with reduced susceptibility, infectivity, and reduced probabilities of transitioning to severe outcomes. The interventions were triggered by time (e.g. school closure) or the global/local state, targeting a defined set of nodes or network edges, and could influence compliance, cause a delay in action, or change the efficacies on node attributes or edge weights.

#### SMH Round 12

**Fig. 3.**
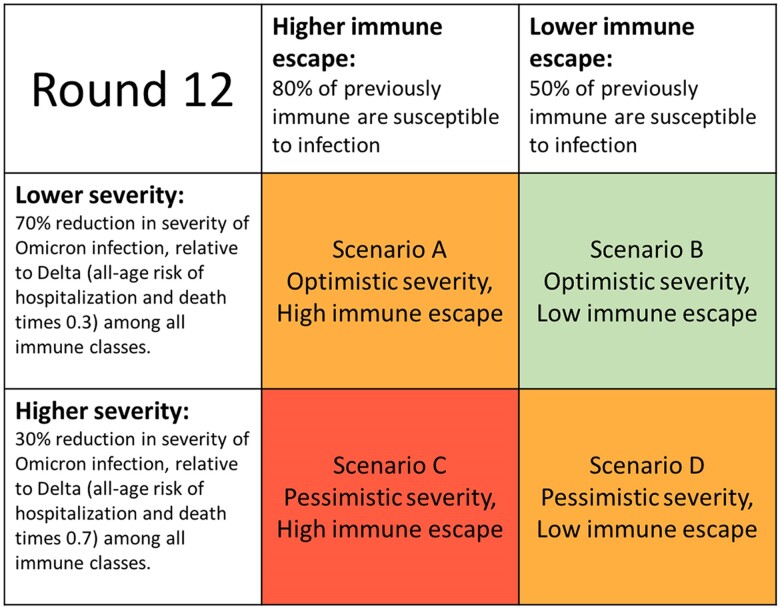
Design of scenarios in CDC COVID-19 SMH Round 12 (55).

Here we showcase how Epihiper was used to support Round 12 of the CDC COVID-19 SMH; additional details regarding this and other rounds can be found at https://covid19scenariomodelinghub.org/archive.html, but see also the Supplementary Material, Section G for a background on the purpose of the SMH. The main purpose of this round, conducted at the beginning of 2022, was to evaluate the impact of the Omicron wave on the disease outcomes in the first three months of 2022 through a design with two axes. (i) The immune escape axis has a higher level of susceptibility, which assumes that nodes with prior immunity (natural or vaccinal) of pre-Omicron variants are 80% more likely to be infected by Omicron than by any pre-Omicron variant; and a lower level of susceptibility, which assumes 50% for this difference. (ii) The severity axis has a pessimistic level, which assumes that the risk of severe outcomes, including hospitalization and death, of a node infected by Omicron is 70% of that had the node been infected by Delta; and an optimistic level, which assumes 30% for the same ratio. Figure 3 shows the 2×2 design with the two axes and four scenarios of Round 12.

**Fig. 4.**
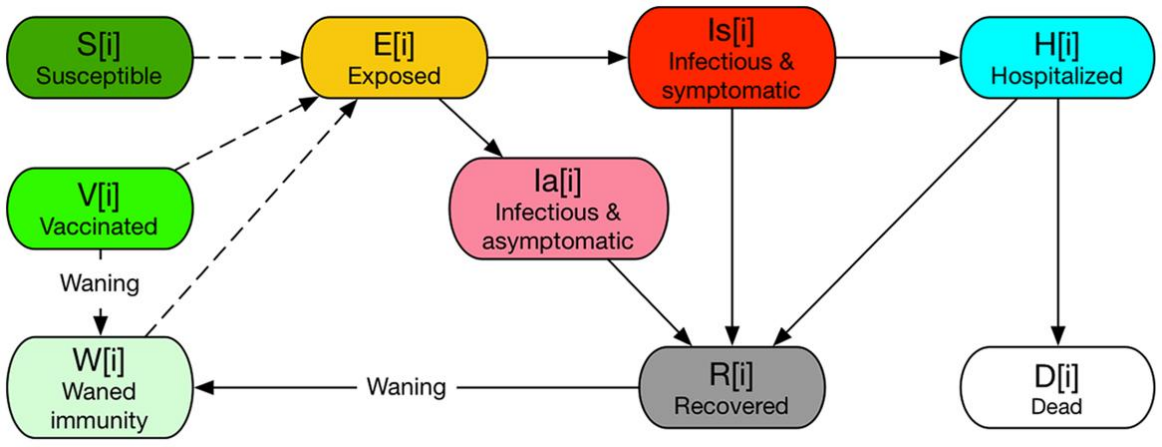
Disease model used for Epihiper simulations in Round 12 of the CDC COVID-19 SMH. Note that the figure shows the states of a node of age group *i*; all age groups have the same states and transitions, but the parameters, including susceptibility, infectivity, dwell time, and transition probability, may be different between different age groups. The states *E*, *I*, and *Ia* are duplicated with the same values of infectivity and susceptibility parameters for Omicron, to keep track of cross-infections between variants due to immune escape.

The Epihiper disease model included the following age-stratified health states: susceptible, vaccinated, exposed, symptomatic, asymptomatic, hospitalized, recovered, and dead, and modeled two COVID-19 strains: the first representing all pre-Omicron variants, and the second corresponding to Omicron. We modeled immunity waning explicitly in the disease model with a partially susceptible state. A node in either recovered state (natural immunity) or vaccinated state (vaccinal immunity) is moved to the partially susceptible state after a random dwell time, sampled from an exponential distribution with a median of 6 months (56). Protection against infection for nodes in the partially susceptible state, as compared with nodes in the fully susceptible state, is 60% for those under 65 years, and 40% for those 65 and older; protection against severe disease is 90% and 80%, respectively. Figure 4 shows a simplified visualization of our disease model. Note that the actual disease model included age stratification for all health states, as well as characterizations for two virus strains. We also interpreted higher severity (as per the SMH 12 description) as larger probabilities of severe outcomes, including being symptomatic, being hospitalized, and being dead. Specifically, the higher probability of being symptomatic is implemented as larger probability to transition from state *E* to state $I_{s}$.

We modeled the same NPIs in all scenarios and across all states: (i) A fraction (15%) of the population chooses to reduce nonessential activities (e.g. shopping, religion, and other). (ii) All K-12 schools are closed from late December 2021 to the beginning of 2022, and face masks are used in school while schools are open. (iii) A fraction (75%) of symptomatic people choose to self-isolate themselves at home. Here the compliance rates of (i) and (iii) are assumed to remain the same during the projection period, but Epihiper can be configured to model time-varying NPIs if supporting data are available.

The simulations were initialized by data-driven assignment of nodes’ initial health states. Based on county-level surveillance data of prior confirmed cases, age-specific case ascertainment rates, state-level data of prior vaccinations, and waning of immunity (natural and vaccinal), we initialized each individual to one of naively susceptible, vaccinated, partially susceptible (with waned immunity), and nonsusceptible (currently or recently infected) states, depending on whether and when the individual is/was infected and/or vaccinated.

For each state, the transmissibility parameter in our disease model was calibrated targeting the state-level estimated effective reproduction number from the most recent confirmed cases. Epihiper simulations were run for each state separately, using multiple replicates, with outcomes combined to get results of the whole United States. The simulations predicted daily infections, hospitalizations, and deaths. Daily results were aggregated to obtain weekly data, with distribution of projections estimated for each target through the simulation replicates.

**Fig. 5.**
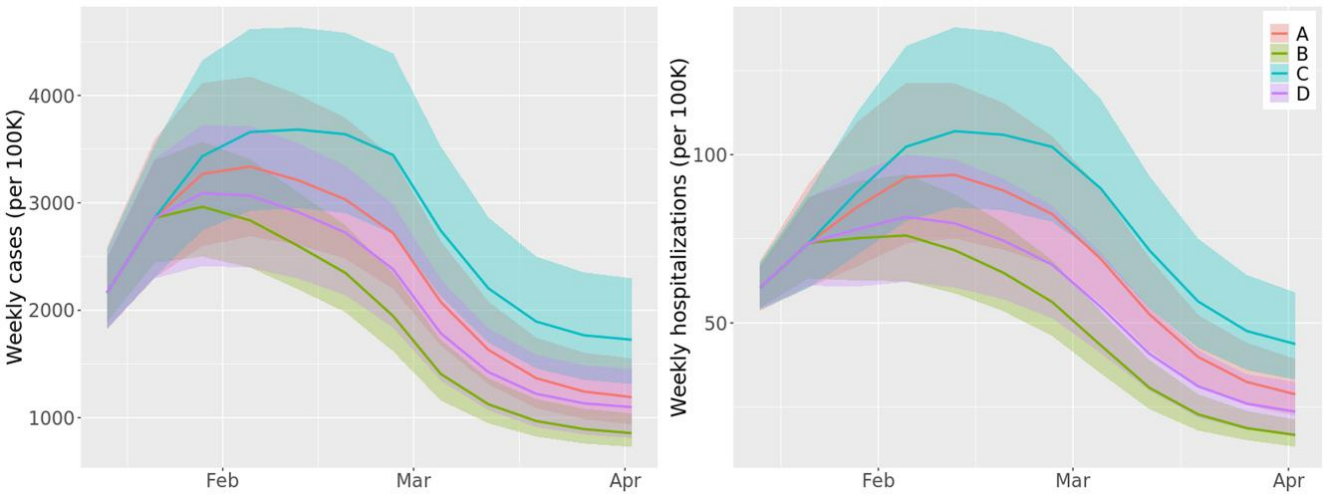
CDC COVID-19 SMH Round 12 projections by Epihiper for cases and hospitalizations under four hypothetical scenarios (A through D from Fig. 3) in the first three months of 2022. The curves show the median of projections, and the ribbons show the 95% projection interval.

At the national level, the Epihiper projections showed that confirmed cases peak in late January (best scenario) to early February (worst scenario), followed by a quick drop to a level that would last for some time. Among the four scenarios, even the best one (B: optimistic severity, low immune escape) may reach 3,000 cases per 100K in the peak week, possibly stressing the testing capacity. Similar trends were projected for hospitalizations, with a less dramatic gap between current and previous peaks compared with cases due to the less severe outcomes of Omicron infections and the protection provided by vaccines. These are shown in Fig. 5. We also projected significant heterogeneities between states regarding peak size and timing in cases and hospitalizations.

For an additional example of how Epihiper has supported the SMH RSV Round 1 at the end of 2023, see Supplementary Material, Section H.

#### Epihiper computational scaling

Details of the implementation and architecture of Epihiper are presented in the Methods section and in the Supplementary material. However, we note that, in order to support the required scaling that handles networks with more than 100 million nodes, Epihiper was designed and implemented to take full advantage of modern HPC hardware. It can distribute an epidemic simulation across as many computational nodes as are available, and, within each node, it can fully use all available cores. Also, a significant effort has gone into *calibration*, and there are several supported approaches. This includes *through parameters*, such as the intrinsic transmissibility, and *disease parameters* and *intervention parameters*, such as compliance and efficacy. In (57), Epihiper was calibrated using a Gaussian process-based, Bayesian framework to fit time series data based on confirmed cases, and, in (54), Epihiper was calibrated to fit an estimated effective reproduction number using the most recent surveillance data.

Rather than only reporting a standard scaling study with large networks and minimal or no interventions, we here considered network size *and* impacts of the disease model and intervention complexities. The detailed analysis for the computational experiment performed on the synthetic population of Virginia (United States) is described in Supplementary Material, Section D with key findings presented here. The study included eight computational experiments (labeled I through VIII) with disease models as specified in Table S2, a collection of interventions as described in Table S3, and the correspondence between experiments and sets of interventions given in Table S4. Each experiment was conducted on compute nodes with dual CPUs having 20 cores each and 375 GB total memory for its specified collection of interventions using 15 replicates.

**Fig. 6.**
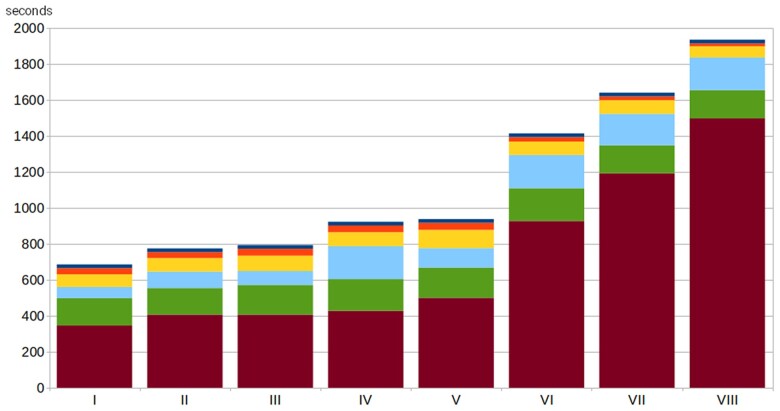
Impact of intervention complexity on computation time for experiments I–VIII (see also Supplementary Material, Section D) factored by the main simulation tasks which are intervention (red bar - segment 1), transmission (green bar - segment 2), update (light blue bar - segment 3), synchronization (yellow bar - segment 4), output (orange bar - segment 5), and initialization (dark blue bar - segment 6). Here segment refers to the block within each bar, with the numbering going from bottom to top. The unit on the *y*-axis is seconds. The experiments were conducted on Intel Xeon 6248 @2.50 GHz processors with 384 Gb of memory, and the population network for Virginia has 7,688,059 nodes and 371,888,620 (directed) edges.

As shown in Fig. 6, the impact of intervention complexity on running time is very clear (indicated by the height of the red-colored bar segment encoding the time spent executing the interventions across experiments I through VIII). In Supplementary Material, Section D, we have also provided Fig. S11 which gives a scaled version analogous to Fig. 6, but in this case taken across all US states using a scenario (different from the above) that was used to support the Scenario Modeling Hub.

**Fig. 7.**
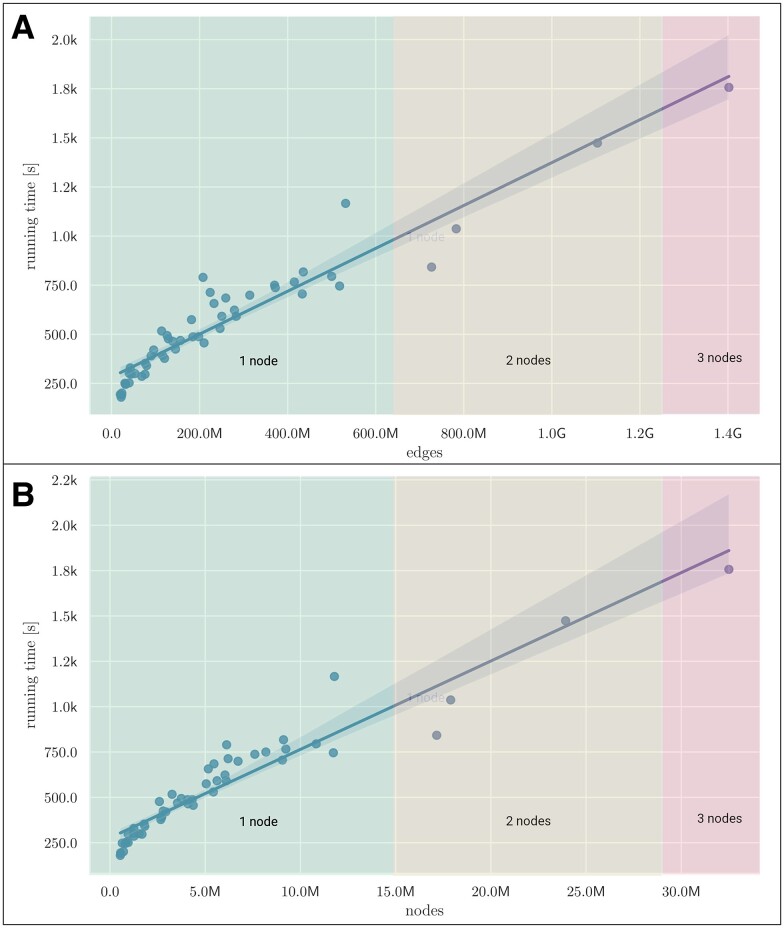
Epihiper running time as a function of network size for the US states with a simulation time of 220 days. Running time as a function of the number of A) nodes and B) edges. All computations have been performed on nodes featuring Intel Xeon 6248 @2.50 GHz processors and 384 Gb memory.

In Fig. 7, we have provided a scaling analysis using the synthetic populations and networks of the US states for one of our vaccine studies (24).

As we see, the running time scales more or less linearly with node and edge counts of the networks.

## Discussion

There are several other frameworks for computational epidemiology. For this discussion, we have focused on **Covasim** (35), **Covid-Sim** (1), **EMOD** (Epidemiological MODeling software) (11), **MATSim-EpiSim** (51, 52, 58), **OpenCOVID** (14), **OpenABM-Covid19** (13), and **FRED** (Framework for Reconstructing Epidemiological Dynamics) (28). Below, we provide a brief overview of these systems; more details, as well as descriptions of some other systems, are provided in the Supplementary material. Following this, we summarize key features of these systems in Table 2, after which we discuss some of the unique aspects of Epihiper as well as elements considered for further development.

**Table 2.** An overview of some key aspects of epidemic simulation models/frameworks.

Framework	Epidemic model	Interventions	Networks	Language	License	Scaling/Parallelization
Covasim (35)	S + C	M + C	SynthPop + random graphs	Python	MIT	Single node/single core
Covid-Sim (1)	S + C	M + C	Layered networks	C++	GPLv3	Single node/multiple cores
FRED (28)^a^	M + C	M + C	Location-based with uniform mixing per location	C++	Custom	Single node/multiple cores
MATSim EpiSim (52, 58)	S + C	M + C	Location-based w/ sub-locations and random graphs	Java	GNU Affero GPL	Single-node/multiple cores
OpenCovid (14)	S + C	M + C	POLYMOD-calibrated random network	R	GPLv2	Single node/single core
OpenABM-Covid19 (13)	S + C (multiple, independent strains)	M + C	Layered, POLYMOD-calibrated, structured random networks + user-provided	C	GPLv3	Single node/multiple cores
EMOD (11)	M + C	M + C	Network-based, patch model (nodes-locations/edges-migration)	C++	CC BY-NC-SA 4.0	Multiple nodes/single core
Epihiper	M + C + P	M + C + P	General, directed, labeled networks	C++	MIT	Multiple nodes/multiple cores

The columns “Epidemic model” and “Interventions” indicate whether (a) a single [S] or multiple [M] versions are provided with the system, (b) if the versions captured in (a) are configurable [C] through a configuration file, and (c) whether new elements can be defined and parameterized externally [P] without having to add and integrate new source code. The “Networks” column indicates what types of networks are supported, “Language” specifies the main programming language used for the system (not that of helper tools), and “License” gives the applicable license. Finally, “Scaling/Parallelization” details whether *a single instance* of the application can be distributed across multiple compute nodes and/or cores.

^a^There is a commercial, closed version of FRED that is owned, maintained, and updated by the company Epistemix.

By software design, the current version of Covasim only supports epidemic runs on a single core. However, the framework can run multiple independent model instances distributed across the cores of a single computing node (using the Python sciris library). We note that Covasim supports dynamic rescaling where an agent in the simulation represents *N* (the scaling factor) individuals, and may thus permit additional scaling for scenarios where this is appropriate, see also (43). Covid-Sim is a threaded application, and may therefore use all the cores on a single computing node. Its software design thus provides inherently better computational scaling than that of Covasim. However, for both Covasim and Covid-Sim, being limited to a single compute node (CPU), there is currently no meaningful way to compare their scaling properties (e.g. with respect to network size) to those of Epihiper. This becomes even more evident for the complex classes of interventions typically needed when supporting the scenarios posed by policymakers. Such interventions frequently require a large set of static and dynamic attributes for each person in the simulation model. This imposes a large memory footprint, severely limiting single-node architectures. OpenCOVID (14) is an R-based epidemics framework focused on SARS-CoV-2 transmission (including strain sub-types) and includes a rich set of configurable pharmaceutical and NPIs. Contact networks for transmission are generated to match contact rates by age-groups using those of POLYMOD (59, 60). The framework provides the functionality to distribute the (single-threaded) simulation instances of calibration processes and computational experiments across a computing cluster. EMOD (11) is a framework for epidemiology supporting a large number of built-in disease classes rooted in discrete stochastic SEIRS models and their sub-classes like SIR, SEIR, and SIS. This includes vector-borne diseases along with representations of environments, and interventions (or *campaigns*, in EMOD terminology). The model is network-based, where nodes represent locations, and it is up to the modeler to select resolution within a node. Edges are captured through the notion of migration, which is used to capture the probabilistic relocation of entities between nodes. Existing disease models and interventions can be parameterized through the use of JSON (JavaScript Object Notation) configuration files. The EMOD code base is written in C++, and exhibits excellent software practices. Thus, when a user needs models or interventions not natively available in EMOD, there is a clearly outlined process for incorporating new functionality (written in C++), including test procedures and support from the EMOD team. EMOD mainly targets MS Windows architectures (with some support for Linux), and can distribute an instance across nodes of a cluster, but does not appear to be using threading. The MATSim-EpiSim model (52, 58) combines a location-based epidemic model for COVID-19 and the powerful MATSim framework (51) that can provide highly realistic, individual mobility traces, integrating, e.g. detailed mobile phone data. The Java-based simulation model was used to advise the German federal government on a range of NPIs throughout the COVID-19 epidemic. Its delineation of epidemic dynamics into disease infection and progression is similar to Epihiper. For infection, a Wells–Riley type model incorporates factors such as viral shedding rates, contact intensity, intake, contact duration, location/room sizes/volumes, and air exchange. The model currently uses a pair of mobility sequences (weekdays, weekends) which are run repeatedly. Contacts within locations are inferred through (i) a partition of visits into sub-locations followed by (ii) a uniform, random inference of edges within sub-locations, a process that is repeated each day (the nonfrozen model). Incorporation of location characteristics at this scale is novel (parameter estimates for the various location type are informed by the literature), as is the inclusion of public transportation captured in the mobility sequences, and the use of temperature as a criterion for classifying activity types such as leisure as indoors/outdoors, the latter reflecting surveyed behavior in Germany. As reported in (52), a single-threaded simulation for Berlin (all 5 million people) took 1 h per simulated month; the simulation model has since been made thread-parallel (58). The OpenABM-Covid19 computational epidemiology framework (13) has a configurable, fixed COVID-19 model, but can capture scenarios with multiple independent strains. The individual-based simulation uses a layered network model with contexts home/household, work/occupational, and other/random, and where degrees (or contact rates) are calibrated against age-stratified POLYMOD data (e.g. (59, 60)). The framework also supports custom networks provided by the user. The core OpenABM tool is written in C with interfaces exposed through R and Python. Their meta-population model is implemented in Python using its multiprocessing module, presumably with each process invoking the single-threaded, core OpenABM application. Like EMOD, OpenABM is rooted in good software practices.

Table 2 contains a summary of key features and aspects for the frameworks just described. We remark that, for this table, a model or intervention entry listed as “fixed” means that this feature is hard-coded. While a user of the software may add additional models or interventions, this would necessitate addition of programming source code, which, in turn, requires adequate coding skills in the system’s programming language and sufficient insight into the implementation design.

Since Epihiper is designed to scale by distributing across computing nodes (rather than using just a single node, threaded architecture), we have omitted direct comparisons with the above frameworks on benchmark scenarios; refer to Supplementary Material, Section D for a more in-depth discussion of this.

A network model is “layered” if it is constructed using layers (e.g. household layer, school layer, work layer) where nodes are joined within layers to satisfy, e.g. data from a mixing matrix (e.g. (59–61)); it may be structured according to demographics (e.g. age bins). While projects such as SynthPop (62), which uses the layered network synthesis approach, offer networks that can be explicitly examined, many epidemiological simulation tools construct their networks on the fly, thus making it hard to assess the networks’ properties or adequacy. An epidemic model is *location-based* if it tracks people’s visits to locations, constructs the contacts at each location, and runs the epidemic process over the induced sub-networks (e.g. FRED (28), MATSim-EpiSim (52, 58), and EpiSimdemics (18)).

A central takeaway from Table 2 is that Epihiper is, as far as we know, the only simulation model that cleanly separates source code and specification of disease models and interventions, the latter being provided externally as input files. Other models have several built-in disease models and interventions. The parameterization of these can be set through input files. However, introducing new disease models or interventions will require changes to these other systems’ source code. This aspect of Epihiper’s design was one of our major design considerations. Granted, there are systems, such as EMOD, with many built-in disease models, including support for vector-borne diseases which the current version of Epihiper may not adequately support. However, tight coupling of code, disease models and interventions has challenges. We remark that there are alternatives to our design which use text-based (JSON) specification of disease models and interventions. Other common design patterns include exposing functionality through a library that a user may employ through new source code. Such a library may have an object-oriented design where the user can derive classes, override specific functions in the class interfaces, and through this obtain their intended adaptation and extensions. Dependency injection is another common pattern (63) where software components compliant with prescribed interfaces can be registered and replace default functionality (e.g. the disease transmission model in Epihiper). This pattern is applied within MATSim (64) on which MATSim-EpiSim is based. A design using dependency injection will generally offer more flexibility than a text-based solution, but it will also raise the complexity and level of expertise required from the user. The design decision for Epihiper was motivated by the success of the text-based approach in the systems biology community (i.e. SBML (44)—see also https://sbml.org/documents/publications/), as well as for reasons already discussed.

Stepping back, we believe a key factor in ensuring that the broader scientific community trusts epidemic models is to base the software on a *formal model* (i.e. a precise mathematical or algorithmic description) that is published in full. This serves two essential purposes: it gives the necessary separation of concern between the model and the code, thus helping avoid the common situation where new model features are implemented directly in the software without first carefully updating the formal model. This situation poses serious challenges for validation and code verification since, in this case, the code has become the de facto formal model: earnest model validation has to be done at the level of source code, a fact that also complicates peer publication and review.

Second, it allows us to take a step towards producing reproducible epidemic modeling environments, see (9, 65–67) for further discussion. Computer scientists have advocated for this approach for a long time as they have developed complex software systems. The design of the Epihiper modeling framework and its software architecture adhered strictly to the above principle; both elements are described in the Methods section with additional details provided in the Supplementary material. In particular, its disease models and interventions are cleanly specified, and can easily be shared or reused in a manner that directly supports peer review.

### Computational scaling

As can be seen in Table 2, many of the comparative systems are currently limited to running a simulation instance (i.e. not separate replicates) on a single compute node, although some are able to take advantage of multiple cores of a node. Epihiper was designed with performance as one of its key goals; a concerted effort was made to provide a precise and rich mathematical model while also taking full advantage of current software architectures. Its intervention language (see the Methods section) does not offer all the functionality and constructs that one will find in programming languages like Python or C++, but has so far been able to fully support all requirements in our work with federal and state agencies. The design of the intervention language, which conceptually has common elements with the structures used in EMOD, was specifically focused on computational complexity and run times to ensure adequate scaling. As detailed in the Results section, we have not attempted to assess the maximum network size that Epihiper can support, nor to push computational boundaries. While that certainly has value, the major driver of computational complexity is generally governed by the collection of interventions that are applied. Epihiper has routinely been used for simulation over our California networks, which have more than 30 million nodes and close to 700 million edges. While we may eventually benchmark Epihiper for network scaling using some standard set of interventions, such results may not be of much practical value to policy formation. The demonstrated linear scaling in terms of network size (node/edge counts of Fig. 7) and computer memory (Fig. S10) leaves us confident that we can scale to the complete US population and beyond.

### Technology choices

The Epihiper simulation model can run on any modern computer ranging from laptops to super-computers. Naturally, larger networks will require matching hardware. The software design uses standard, open technologies, such as MPI (68) and OpenMP (69) for computing, PostgreSQL for the person trait database, and Frictionless (70) and JSON formats for specification of input data. This ensures that it can be deployed virtually anywhere.

### Limitations

Here we list Epihiper limitations organized into categories.

#### Limitations regarding model generality

The Epihiper programmable disease- and intervention model primarily targets diseases transmitting directly from human to human. The ability to accurately capture, e.g. vector-borne diseases or diseases that spread via inanimate objects is limited. Epihiper does also not directly model factors such as individual antibody levels across strains. Regarding input data, the population- and network data used with Epihiper has been limited to the case of a repeating, daily network representing a typical weekday. Actual contact network would likely have a combination of repeating and random contacts, examples being contacts at home and at work (repeating) while contacts taking place at, e.g. retail locations would have a large degree of variability. Yet more realistic contact networks model could incorporate seasonal fluctuations where, for example, contacts during leisure-type activities are more likely to occur outside during summer months. Epihiper and its input location data only consider location type (e.g. residence, school, place of worship) and do not explicitly model factors such as building air-flows and ventilation systems. Unlike MATSim-EpiSim, transmission occurring during transit between activities (e.g. public transportation) are also not included. Dynamic aspects of Epihiper’s input networks are limited to those that arise through interventions such as school closures. Finally, Epihiper’s intervention language was designed for epidemics, and is not a general programming language. The correctness of the Epihiper implementation is tied to the time-horizon condition of Theorem 1 along with restrictions on unattached variables. These must be carefully observed to ensure valid use.

#### Limitations regarding operation

For advanced use, such as supporting public health agencies during an epidemic, Epihiper generally requires a team with a broad set of expertise areas. This includes trained modelers and practitioners who can map epidemiological aspects and the literature into disease models and realistic interventions. Moreover, encoding these in their JSON representations will require experience with this standard, especially when operating under short turnaround times. Scenario planning will typically require experience developing experimental designs to support a range of conditions as well as sensitivity analyses and uncertainty quantification. Computer engineering expertise will be required to install and configure Epihiper, especially when it is run on an HPC architecture such as a cluster. Execution of simulation runs associated with experimental designs, the data management, and the analysis, require a combined expertise of computer engineering, data management, statistical analysis, and general data analytics.

#### Limitations regarding software architecture

Epihiper uses external, text-based specifications of disease models and interventions. Other software patterns include (i) offering Epihiper as a software library and/or (ii) employing a design that supports dependency injection (63) at specific points of an overall computational workflow. This can be an attractive option to advanced users seeking to extend Epihiper functionality that somewhat lessens the need to fully understand the entire code base. The Epihiper architecture uses a multinode, multicore design. Most other simulation models in this field (e.g. the ones listed in Table 2), employ a single-node, multicore design. For problem sizes that fit within the memory of a single computing node, the latter category of systems will generally scale better as measured by processing time. For a brief discussion of issues and challenges that arise when comparing these two classes of models, see the Supplementary Material, Section D.

#### Limitations regarding computing resources

For scenarios involving large populations and networks, Epihiper will typically require HPC hardware having multiple computing nodes with significant memory, a resource that many may not have access to. Similarly, scenarios involving large experimental designs may only be feasible with access to a large number of computing nodes as their sequential execution is too time-consuming.

#### Limitations related to input data

For an epidemics scenario, Epihiper relies on input data capturing the population and its contact network for the region in question. Such data are not widely available, a fact that may eliminate one’s options regarding the use of Epihiper. While one may construct basic versions of such data, perhaps through a combination of demographic census data and random graph approaches, ensuring adequate data fidelity and precision is a challenge. Collecting and harmonizing the wide diversity of data that goes into their construction, calibration, and validation (as well as the actual computation and data management aspects) adds to the challenge.

### Summary

The computational scaling exhibited by Epihiper may not be necessary for many studies for which smaller or reduced-order models are sufficient. However, as public health questions become more detailed, with interventions and priorities targeting specific sub-demographics at a larger scale, the need for computational tools like Epihiper will increase. Questions that address early stage epidemics (e.g. introduction of a novel disease or strain to a continent), where it is unclear a priori whether a reduced-order model is sufficient, represent an additional argument for highly detailed models. A highly detailed model can deal with these situations directly and may be used to determine what levels of model reduction are acceptable in a given situation. This is particularly true for scenarios where there is no historical data. In closing, we believe that the role of simulation-based reasoning in support of public health and policy formation will become increasingly more central, and we anticipate that many of the limitations and challenges that apply to Epihiper and other epidemic simulation models will continue to be addressed and resolved over the coming years

## Materials and methods

### Software design and implementation

The Epihiper software architecture is a hybrid MPI/OpenMP design, and is implemented in C++ for high performance. (Here *software architecture* means the description of the software’s components, the dependencies among the components, and their composition into subsystems, including structural approaches used for parallelization.) The contact networks can be represented either as text or binary files, with the option to perform prepartitioning for the desired target combinations of compute nodes and cores. Customizable traits can be attached to each person and each edge, and are exposed to Epihiper through a software PostgreSQL database. This database, which can be shared among computational experiments, has been finely tuned to handle a large number of simultaneous queries, particularly as they occur at the initialization stage of large Epihiper compute jobs. Details regarding the Epihiper software architecture are provided in the Supplementary material.

### Correctness and invariance

We would like to assert that Epihiper’s software architecture (whose diagrams are provided in Supplementary Material, Section C) faithfully captures the Epihiper formal model. Here we describe conditions that guarantee this will hold, and also include an invariance property. First, the Epihiper model and implementation supports variables that are not attached to vertices or edges; we refer to such variables as *unattached variables*. For example, in the case of PIs one may have constrained resources, such as a limited number of vaccine doses. A straightforward way to implement this is to introduce an Epihiper variable (e.g. vaccineCounter) that tracks the count of remaining doses and that is decremented whenever a dose of this vaccine is administered. In a multiprocess simulation for Epihiper, the computing nodes (CPUs) generally operate at different speeds (e.g. due to differences in loads). Clearly, a process assigned to a faster node may lay claim to and possibly exhaust resources (e.g. vaccines) before processes on a slower node can do so.

For performance reasons, Epihiper’s software architecture assumes that the diseases being modeled are such that the time scale for transitioning into an infected state is at least that of a time step (called a tick in Epihiper). In other words, the diseases are such that no person can enter an infectious state in the middle of a time step and subsequently go on to infect others in the remaining part of the time step. We note that this condition can generally be met by adjusting the amount of time covered by a tick. We call this the *infection time condition*.

With these clarifications and the Epihiper software architecture as detailed in the Supplementary material, we can now state:

Theorem 1 **(Correctness and invariance)**

1. When invoked on a disease model and a set of interventions that does not contain unattached variables, and when configured such that the infection time condition holds, the Epihiper software architecture correctly implements the Epihiper formal model.
2. Modulo the equivalence on orders of operations of equal priority, two Epihiper instances applied to the same input specification generate the same output.

Regarding the second statement (invariance), it is understood that the input specification includes (i) the random seed(s) used to initialize random number generators, and (ii) the process specification (i.e. the number of compute nodes).

### Synthetic populations and contact networks

Epihiper uses the notion of a digital twin of the population and a social contact network of a region to describe the people (vertices) and their interactions (edges), see (71). The synthesized population consists of a base population partitioned into households with demographic attributes (e.g. age, gender, worker status, NAICS (North American Industry Classification System) code, and household attributes such as household income. These attributes are provided to Epihiper via its person trait database. Based on demographic attributes, each person is matched with a detailed activity sequence (e.g. from NHTS (72)) covering their activities on a typical day, and each such activity is assigned a location using an algorithm outlined in the Supplementary material.

**Fig. 8.**
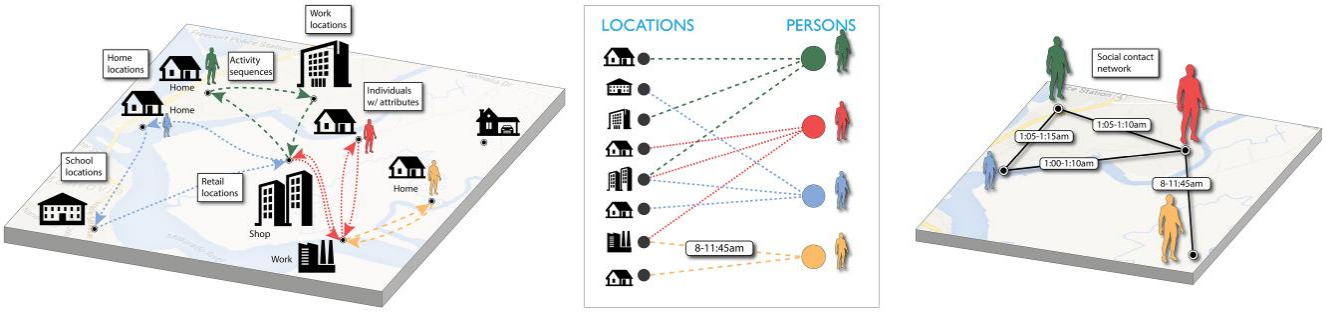
An illustration of the population and network components used in Epihiper. Location assignment is illustrated on the left, and captured formally as the people-location network $G_{PL}$ (middle), which, in turn, gives rise to a social contact network $G_{P}$ (right).

Using the assignment of all people’s activities to locations (which we refer to as the person-location graph $G_{PL}$), we construct a social contact network $G_{P}$ with nodes $V_{P}$ (people) and edges $E_{P}$ inferred through a per-location contact model adapted from the Erdős–Rényi random graph. The adaptation is designed to bound the maximal degree at each point in time at that location through its model parameters. The common approach that incorporates edges for all simultaneous visits can easily lead to unrealistically large vertex degrees, with potential challenges for calibration through a global transmissibility parameter, see the Supplementary Material, Section F. Each node $v\in V_{P}$ has a number of configurable attributes specific to the questions being studied; similarly, each edge $e\in E_{P}$ has an associated set of configurable attributes that includes (at minimum) the target (resp. source) person ID, the target (resp. source) person activity type, and the duration of contact. The main components of the synthetic population and its network are illustrated in Fig. 8 with additional detail provided in the Supplementary material.

## Supplementary Material

pgae557_Supplementary_Data

## Data Availability

All data sets used for Epihiper examples and tutorials (e.g. population data, networks, disease models, and interventions) are provided at https://net.science/files/resources/epihiper/examples/ under the data license terms specified in the metadata. Epihiper examples illustrating its use can be found at https://epihiper.readthedocs.io/en/latest/examples/examples.html. Each example lists the URLs to the required data sets (all located under the above URL). In particular, this includes the detailed example described in Supplementary Material, Section E. In addition, we have made available a small population and network that can be used for very basic testing of Epihiper. These data are distributed as part of the main Epihiper git repository https://github.com/NSSAC/EpiHiper under ./tree/main/example relative to the root of the repository. The Epihiper code base can be accessed at https://github.com/NSSAC/EpiHiper and is made available under the MIT license https://mit-license.org/. Installation instructions are provided in https://epihiper.readthedocs.io/en/latest/quickstart/get-started.html with the main documentation provided at https://epihiper.readthedocs.io/en/latest/index.html.

## Notes

a By reproducibility, we mean the ability for other researchers to re-implement the model using knowledge obtained from the original paper, possibly in a different simulation tool or programming language than the one reported, and, through simulation, verify the results reported in the study (41).

b
https://covid19scenariomodelinghub.org/

c Information about our ongoing efforts to support these agencies can be found at: https://nssac.github.io/covid-19/index.
