# Supplementary material for: Epihiper—A high performance computational modeling framework to support epidemic science
Source: PNAS Nexus. 2024 Dec 11;4(1):pgae557. doi: 10.1093/pnasnexus/pgae557 (PMC11667244; doi:10.1093/pnasnexus/pgae557)
Supplement: pgae557_Supplementary_Data [file pgae557_supplementary_data.pdf]

Supplementary Information for

EpiHiper - A High Performance Computational Modeling Framework to Support Epidemic Science

Authors:

Jiangzhuo Chen, Stefan Hoops, Henning S. Mortveit, Bryan L. Lewis, Dustin Machi, Parantapa Bhattacharya, Srini Venkatramanan, Mandy L. Wilson, Chris L. Barrett, and Madhav V. Marathe

Corresponding author:

email: henning.mortveit@virginia.edu

This PDF file includes:

Supplementary text
Figures S1 to S14
Tables S1 to S4
SI References

Supplementary Material

A. The EpiHiper model. This section covers all aspects of the EpiHiper model starting from foundational concepts and going all the way through interventions. Examples of all concepts are provided in Supplementary Material Section B.

A.1. Core concepts. **Time and Iterations.** The model advances in discrete time steps t_0, t_1, t_2, ..., t_n starting at t_0 and where *iteration k* advances time from t_{k-1} to t_k. The duration of each iteration is constant and is denoted by T, which implies that $t_n = t_{n-1} + T$. Iteration 0 handles all initialization and anything that takes place up until the time t_0. In particular, this covers invocation of interventions, including vaccinations that have been administered to members of the population prior to t_0.

Health states. The disease model has a set of *health states* denoted by

$$\mathcal{X} = \{X_1, X_2, \ldots, X_m\} . \qquad [2]$$

For a standard SIR-model, we have $\mathcal{X} = \{S, I, R\}$. Each person, represented as a network node, is assigned a time-varying *health state* that is updated at initialization and as the simulation progresses through the *transmission process* and the *disease progression*. Health states can also be updated during interventions.

Susceptibility and infectivity. Each health state $X \in \mathcal{X}$ has associated *susceptibility* and *infectivity* denoted by $\sigma(X)$ and $\iota(X)$. These are fixed disease parameters (and are thus independent of people). To model *susceptibility* and *infectivity* of *individuals*, which will generally depend on factors such as vaccination and use of personal protective equipment, we assign each person P an *infectivity scaling factor* and a *susceptibility scaling factor*, and denote these factors by $\beta_\iota(P)$ and $\beta_\sigma(P)$. These are all time-varying with a default value of 1.0. Following this, the *effective susceptibility and infectivity* of person P in health state X_i are modeled as (skipping the reference to time, i.e., t_n)

$$\sigma_P(X_i) = \beta_\sigma(P) \times \sigma(X_i) \text{ , and}$$
$$\iota_P(X_i) = \beta_\iota(P) \times \iota(X_i) \text{ .}$$

Traits. EpiHiper supports the notion of *traits*. These are configurable, dynamic variables that can be attached to each person (node traits) and/or to each contact (edge traits). The collection of traits are part of the model specification, and are one of the foundations for interventions, which we present below.

An example of a node trait useful to scenarios involving vaccination is a Boolean flag `vaccinated` that (through interventions) can track a person's vaccination status. Similarly, an edge may have an associated Boolean edge trait `indoors` encoding if the contact takes place indoors. An intervention can then conditionally handle people of different vaccination statuses, as well as indoor and outdoor contacts.

Variables. In addition to the per-person or per-contact traits described above, EpiHiper supports declaration of *variables*. A prime example of a variable could be the number of vaccine doses administered within a health district on any given day, thus ensuring that the model can handle constrained resources. Another example of a variable is one tracking whether school closures are in effect. We note that variables are updated as part of interventions, and also that EpiHiper tracks a collection of standard observables, such as the total and relative counts of people in each of the health states as defined by the disease model.

EpiHiper primitives. These are the basic quantities that EpiHiper makes available for operations, either as part of the right-hand-side expression of assignments or as the left-hand-side (often referred to as an `lValue`) in assignment operations. Table S1 provides a list.

Sets. EpiHiper supports the declaration of sets. Sets may contain nodes and edges, and are formed using standard combinations of logical predicates, variables and EpiHiper primitives. An example usage of sets is to pre-compute and cache frequently used population subsets as intervention targets. Sets are a special case of variables. Section B of the Supplementary Material provides an example related to school closure and vaccinations.

A.2. The EpiHiper disease model. The disease model is split into (*i*) *disease transmission* and (*ii*) *disease progression*. The transmission process governs how individuals become infected, while the disease progression captures the health state evolution once infected. While it may be convenient to combine these processes, it is important to note that they are structurally different: transmission requires the presence of one or more infectious people to infect a candidate susceptible person.[**]

Disease Transmission. Disease transmissions that arise from contacts between infectious and susceptible individuals are modeled as follows: first, contacts are captured as directed edges in the contact network (see Section F of the Supplementary Material). The potential infections that may take place are determined through a person's incident edges and a list of *contact configurations* of health states. Specifically, an individual P in health state X_i (the *entry state*) may transition to state X_j (the *exit state*) when in contact with a person P' in state X_k (the *contact state*). We call this a *transmission configuration*, denote it by $T_{i,j,k} = T(X_i, X_j, X_k)$, and associate to it the *transmission weight* $\omega_{i,j,k} = \omega(T_{i,j,k})$. This weight represents the relative weight of this particular transition, and is set to 1 by default. For modeling the infection process, one will specify all the possible transmission configurations. Note that transmission configurations are disease parameters and independent of people and their attributes. In accordance with standard terminology, we call any entry state a *susceptible state*, any exit state an *exposed state*, and any contact state an *infectious state*. An EpiHiper model may have multiple susceptible and infectious states.

For the infection (or transmission) process of a person P in susceptible state X_i, we need to consider all possible transitions from X_i to possible exit states X_j in the presence of persons P' in possible contact states X_k.

Next, a disease model will have a *transmissibility* that we denote by τ. It is a global parameter representing a rate proportional to the likelihood of becoming infected by being in contact with a single infectious individual for one time unit; it may be used for calibration of, for example, $R_{\text{effective}}$.

Finally, each edge e in the contact network is of the form

$$e = (P', a(P'), P, a(P), w, \alpha, T, \text{edgeTrait})$$

where $a(P')$ is the *activity* of person P' at the time of contact, T is the contact duration (within the current iteration), w is an edge weight, and α is Boolean (edge) variable indicating whether or not the edge is active. Finally, an edgeTrait is a configurable (but optional) set of variables (edge traits) that may have been encoded when constructing the network. Figure S1 provides an overview of all the parameters and concepts covered above and that are involved in the transmission process.

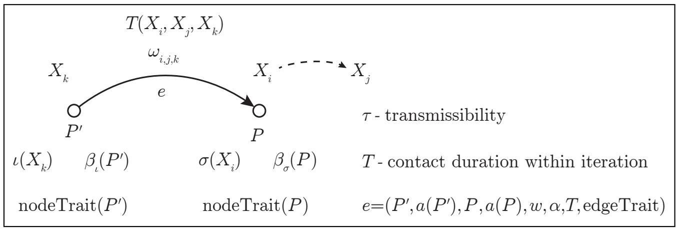

Fig. S1. Overview of disease model parameters governing disease transmission.

Under the assumption that transmissions across all edges and contact configurations are independent for each person P and their contacts P', we define the *propensity* ρ for the edge e and contact configuration as:

$$\rho(P, P', T_{i,j,k}, e) = \Big[T \cdot \tau\Big] \times w_e \times \alpha_e \times \qquad [3]$$
$$\Big[\beta_s(P) \cdot \sigma(X_i)\Big] \times \Big[\beta_i(P') \cdot \iota(X_k)\Big] \times \omega(T_{i,j,k})$$

The algorithm to determine whether a disease transmission takes place in the current iteration t_n with length Δt_n (and also

[**] EpiHiper does not currently incorporate transmission through inanimate objects.

```
-----------------------------------------------------------------------------------------
  node.beta_i                        |rw|   # infectivity scaling factor
  node.beta_s                        |rw|   # susceptibility scaling factor
  node.healthState                   |rw|   # health state
  node.nodeTrait[featureName]        |rw|   # nodeTrait featureName of node
  edge.edgeTrait[featureName]        |rw|   # edgeTrait featureName of edge
  edge.active                        |rw|   # active flag of edge
  edge.weight                        |rw|   # weight of edge
-----------------------------------------------------------------------------------------
  node.id                            |r |   # PID of node
  edge.sourceActivity[featureName]   |r |   # activiy of source of edge [only activityType]
  edge.targetActivity[featureName]   |r |   # activity of target of edge [only activityType]
  edge.sourceID                      |r |   # source vertex ID of edge
  edge.targetID                      |r |   # target vertex ID of edge
-----------------------------------------------------------------------------------------
  observable                         |r |   # current time, total population, etc
  transmissibility                   |rw|   # global transmissibility
  <SetName>                          |r |   # any set referenced by name
  <VariableName>                     |rw|   # any variable referenced by name
  <TriggerName>                      |r |   # any trigger referenced by name
-----------------------------------------------------------------------------------------
```

Table S1. EpiHiper 1.0 primitives; here `w` means writable and `r` means readable.

the decision of which contact to attribute the transmission) as a result of all candidate transmission configurations is modeled after the Direct Gillespie Method (48, 49).

For $(m \geq 1)$ transmission configuration candidates for person P within an iteration, we choose the actual contact configuration (i.e., person P' and contact configuration $T_{i,j,k}$ to whom we attribute the infection) by calculating

$$A(P) = \sum_{P',j,k} \rho(P, P', T_{i,j,k}) \ , \qquad [4]$$

where the sum extends over all neighbors P' of P and indices j and k for which a transmission may occur. We remark that the form of $A(P)$ is structurally very similar to what is used in MATSim-EpiSim (52, 53), although we do not include factors for air circulation and room capacity. Without loss of generality, we assume that the index set of triples $K = \{(P', j, k)\}$ is well ordered. To determine *if* we have a transition, we sample a random number

$$a = -\ln(\text{uniform}(0,1))/A \ , \qquad [5]$$

and, if the inequality $a \leq \Delta t_n$ holds, we select the actual transition by sampling a uniform random number $\alpha \in [0, A]$ and determine the index $\kappa \in K$ for which

$$\sum_{\kappa-1} \rho(P, P', T_{i,j,k}) < \alpha \leq \sum_{\kappa} \rho(P, P', T_{i,j,k}) \ . \qquad [6]$$

Finally, the selected health state transition is scheduled for time t_n. We remark that this is also referred to as a *dose-response model* in the literature (50, 51). The EpiHiper approach using propensities (or the equivalent form in MATSim-EpiSim (53)) is quite flexible, and can be readily extended to incorporate other factors, one example being per-person immunity modeling directly in terms of antibody levels across strains as in (51).

Disease Progression. The disease progression process covers the health state transitions within an individual P that are independent of other people. For the EpiHiper model, a disease progression diagram (or specification) describes all the possible health state transitions that may take place within a person in the absence of transmission processes and interventions. The diagram has the set of health states $\mathcal{X} = \{X_i\}$ and directed edges of the form $e = (X_i, X_j)$, each edge with an assigned probability $p_e = \text{prob}(X_i, X_j)$ and a *dwell time distribution* D_e. We require that, for each state X_i, the sum $\sum_j \text{prob}(X_i, X_j)$ over outgoing edges must equal 1. The dwell time distribution D_e for $e = (X_i, X_j)$ is the probability density for the dwell time in health state X_i given that the transition $X_i \longrightarrow X_j$ takes place.

Algorithmically, disease progression is modeled as follows: when an individual P enters a state X_i, the next state X_j is sampled according to the next state distribution induced by the probabilities p_e, the dwell time ΔT in state X_i is determined by sampling the dwell time distribution D_e, rounded to the nearest integer if necessary, and then bounded below by 0. Finally, the health state transition is scheduled to take place at iteration $t_n + \Delta T$. We remark that disease progression may be overridden by interventions and transmissions, should such events occur before the scheduled health state transition dictated by the disease progression.

A.3. Interventions. Disease transmission and disease progression governs the dynamics of health states in EpiHiper. However, the real power of EpiHiper lies in its rich *intervention model*. An intervention $\mathcal{I}$ is a triple $\mathcal{I} = (T, E, \mathfrak{O})$ where T is the *trigger condition*, a Boolean expression; the set E is the *intervention target*, a collection of vertices and edges; and $\mathfrak{O}$ is a set of operations that can be applied to the variables associated with the elements of target set E, as well as global variables. The trigger expression is a Boolean expression involving EpiHiper primitives and possible sizes of sets. An intervention is executed for every iteration for which its trigger evaluates to `true` at the start of the iteration.

While expressiveness of the set of operations $\mathfrak{O}$ of an intervention may not fully rival that of a general purpose programming language, the constructs that it permits are quite powerful. To describe this, we use some new notions:

- `operation`: an assignment to a variable of the system;
- `operationList`: an ordered, possibly empty, list of `operation`s;
- `operationEnsemble`: the permissible constructs used with EpiHiper.

The recursive, grammar-like definition of what EpiHiper accepts is shown below:

```
operationEnsemble :=
  once
    <operationList>
  foreach
    <operationList>
  sampling <samplingSpecification>
    sampled
      <operationEnsemble>
    nonsampled
      <operationEnsemble>
```

where

```
samplingSpecification :=
(
  relativeSampling ( individual | \
```

```
    group ) <percentage> |
  absoluteSampling <integer>
)
```

and

```
operationList := <operation>+

operation := <variable>
   <operator> <expression> \
     delay(<integer>)      \
   [ priority(<integer>) ] \
   [ condition(<bool_expression>) ]

operator := ( = | *= | /= | += | -= )
```

The blocks within the `actionEnsemble` have the following semantics:

`once`: the `operationList` of the `once` block will be executed precisely once when the intervention is executed. Its purpose is to serve as a mechanism to invoke actions just once if the intervention is triggered to assign, e.g., variables.

`foreach`: the `operationList` of the `foreach` block will be executed once against each element of the target set. If the target element is a node (resp. edge), only action statements that are valid for nodes (resp. edges) and variables will be executed while edges (resp. nodes) will be ignored.

`sampling`: the `sampling` block first partitions the target set into two sets called `sampledSet` and `nonSampledSet` where (using set notation) $\texttt{nonSampledSet} = \texttt{target} \backslash \texttt{sampledSet}$. The set `sampledSet` is determined as follows:

- `absoluteSampling N`: samples precisely $\min(\texttt{N}, |\texttt{target}|)$ elements of the target set;
- `relativeSampling individual percentage`: each element of the target set is sampled with probability `percentage/100` to form `sampledSet`;
- `relativeSampling group percentage`: precisely `percentage` percent of the target set is sampled at random to form `sampledSet`.

The target set of the `sampled` branch is `sampledSet`, and the target set of the `nonsampled` branch is `nonSampledSet`.

Requirement: at least one of the `once`, `foreach`, and `sampling` blocks must be present to form a valid `actionEnsemble`. There is no limit on the recursion depth made available through the sampling control structure, but, in practice, this will not be a concern.

B. Illustration of all EpiHiper model concepts. As an illustration, consider a hypothetical case of a classic influenza (or COVID-like) outbreak in Albemarle County, Virginia with health states

$$\mathcal{X} = \{S, E, Isymp, Iasymp, R\}$$

encoding health states susceptible (S), exposed (E), infectious and symptomatic ($Isymp$), infectious and asymptomatic ($Iasymp$), and recovered (R). A combined transmission and progression diagram for the example is as follows:

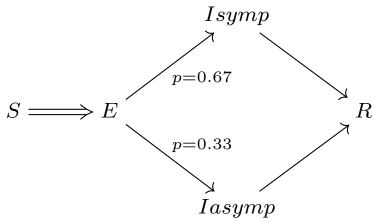

Apart from the double arrow/edge $S \Longrightarrow E$ which specifies transmission (see below), each edge corresponds to a transition in the disease progression model. We note that disease progression from health state E to $Isymp$ is twice as likely as progression from E to $Iasymp$ (or 0.67/0.33, to be precise). The dwell-time distribution for both transitions out of the state E, which we denote by edges $(E, Isymp)$ and $(E, Iasymp)$, are

$$\{0 : 0.1; 1 : 0.2; 2 : 0.6; 3 : 0.1\} ,$$

meaning that, e.g., the probability of a dwell-time of duration 2 (days) is 0.6. Similarly, the dwell-time distributions for the transitions $(Isymp, R)$ and $(Iasymp, R)$ out of states $Isymp$ and $Iasymp$ are both

$$\{3 : 0.3; 4 : 0.4; 5 : 0.2; 6 : 0.1\} .$$

Note that a dwell-time distribution is associated with an edge (health state transition), and that the unit of time is one iteration which in this example equals one day.

The two transmission configurations that arise from the susceptible state (S) and the two infectious states ($Isymp$ and $Iasymp$) are captured as

$$T_s = T(S, E, Isymp) \text{ and } T_a = T(S, E, Iasymp) ,$$

both having the default weight of $\omega_s = \omega_a = 1.0$. Regarding infectivity, people in either of the states $Isymp$ and $Iasymp$ can transmit infections, with the asymptomatic reduction in infectivity being 60% and thus $\beta_\iota(Iasymp) = 0.40$, while the susceptibility and infectivity values of all other health states are kept at their default value of 1.0

(a) School closure. Schools will be closed each day for which 10% or more of the population are infectious (health states $Isymp$ or ($Iasymp$) at the beginning of the day). While one may argue that this is an unrealistic condition (unless there is a very vigorous testing mandate), it will serve for the purpose of illustration. We denote the set of agents by V.
Trigger condition: Using $X(p)$ to denote the health state of person $p \in V$, and N to denote the population size, the Boolean trigger expression C_a can be stated as:

$$\Big|\Big\{p \in V \mid X(p) \in \{Iasymp, Isymp\}\Big\}\Big|/N \geq 0.10$$

Target: for this intervention, the target set T_a is a subset of the edge set E. Writing a (directed) edge as $e = (v, v')$ and denoting by $A_e(v)$ and $A_e(v')$ the activity of v and v' at their time of contact encoded by the edge e, the target set can be expressed as:

$$T_a = \{e \in E \mid A_e(v) = \texttt{School} \text{ or } A_e(v') = \texttt{School}\}$$

Operation ensemble:

```
foreach // Note: "e in T" is implicit
  active = 0, delay(0), priority(0)
  active = 1, delay(1), priority(1)
```

The operation ensemble will deactivate any edge associated with a school activity, and will do so immediately (current day). It will also schedule a school re-opening on the following day by re-activating the edge with a delay of 1. Note, however, that if the trigger condition holds on the following day as well, this intervention will be executed again. In this case, the edge deactivation for the next day will take priority over the edge reactivation that is scheduled on the current day. This illustrates an essential design point: by allowing delays and priorities, we can avoid the bookkeeping of tracking which entities and/or their variables were modified, as well as when they may need to be restored.

(b) Vaccination. In this case, we have a pharmaceutical intervention where people of age ≥ 60 are advised to accept a vaccine offered on day 5, with a compliance rate of 90%, the vaccine being 80% efficacious causing an 80% reduction in susceptibility and infectivity. The vaccine becomes effective two days following inoculation.
Trigger condition: In this case, we simply use the EpiHiper observable `time` to form the Boolean trigger expression as

$$C_b : \texttt{time} = 5 .$$

Target set: In this case, the target set consists of agents of age at least 60:

$$T_b = \{v \in V \mid \texttt{age}(v) \geq 60\}$$

Operation ensemble:

```
sampling relativeSampling individual 90.0
  sampled // sampled subset of T_b
    beta_i *= 0.20, delay(2)
    beta_s *= 0.20, delay(2)
  nonsampled
    {}
```

Listing S1. The EpiHiper initialization

```
Algorithm: InitializeEpiHiper
  if network not partitioned :
    PartitionNetwork()

  LoadAndCompile:
    network, diseaseModel, traits,
    interventions, initialization

  InitializePersonTraitDB()

  t := Tstart − 1
  Initialize:
    Output, StateCounts

  CreateDependencyGraph()
  UpdateAllDependencies()

  Scheduling:
    ProcessInitialization()
  Update:
    ExecuteActions()

  t += 1
  Output:
    healthStateChanges
    healthStateCounts
    network // optional

  SynchronizeChanges()
```

C. The EpiHiper Discrete time parallel simulator.

Architecture description. EpiHiper is a software application designed to work on an HPC system, as well as on a desktop environment. It is implemented in C++ and uses MPI and OpenMP for parallel processing. The need for parallel processing arises mainly from the memory requirement for storing the contact network (vertices and edges) which has time-evolving attributes, but it obviously also has an impact on the compute time. During initialization (see the algorithm in Listing S1), the network is distributed such that each process (i.e., compute node) is assigned a fixed set of vertices and all of their incoming edges in order to ensure that the memory requirements are evenly distributed. For the operations described in Supplementary Information Section A.3, only the owner process of vertices and edges is allowed to update their states. With this restriction, the execution of operations is done by each process using the queuing model described in the main paper's Results section. While processing interventions, variables of vertices or edges belonging to other processes may need to be altered. This is handled by scheduling these operations on the processes (i.e., compute nodes) that own those vertices or edges. In particular, we note that the disease process (transmission and progression) of vertices is fully controlled by their owner process.

As can be seen in the algorithms of Listings S1 and S2, we achieve unambiguous processing by cleanly separating the point of execution of operations from the point where operations are scheduled through disease transmission, disease progression, or by interventions.

A note on health state updates. Since it may not be obvious, we remark on how health state changes are implemented and executed: upon entry to a new health state X, the successor state X' is immediately determined along with the dwell time t_d in health state X'. The operation that sets the health state to X' is then scheduled with a delay of t_d and with suitable priorities and conditions as needed.

Detailed algorithms. The following diagrams and figures detail the main algorithm and expansions thereof as per the following list:

Listing S2. The EpiHiper main algorithm

```
InitializeEpiHiper()

while t < Tend :
  ResetVariable()
  UpdateAllDependencies()
  ProcessTriggers()

  Scheduling:
    InfectionProcess()
    ProcessInterventions()
  Update:
    ExecuteActions()

  t += 1
  Output:
    healthStateChanges
    healthStateCounts
    network // optional

  SynchronizeChanges()
```

- Main process: Figure S2
- Transmission processing: Figure S3
- Trigger processing: Figure S4
- Intervention processing: Figure S5
- Action ensemble processing: Figure S6
- Action scheduling processing: Figure S7
- Action execution processing: Figure S8

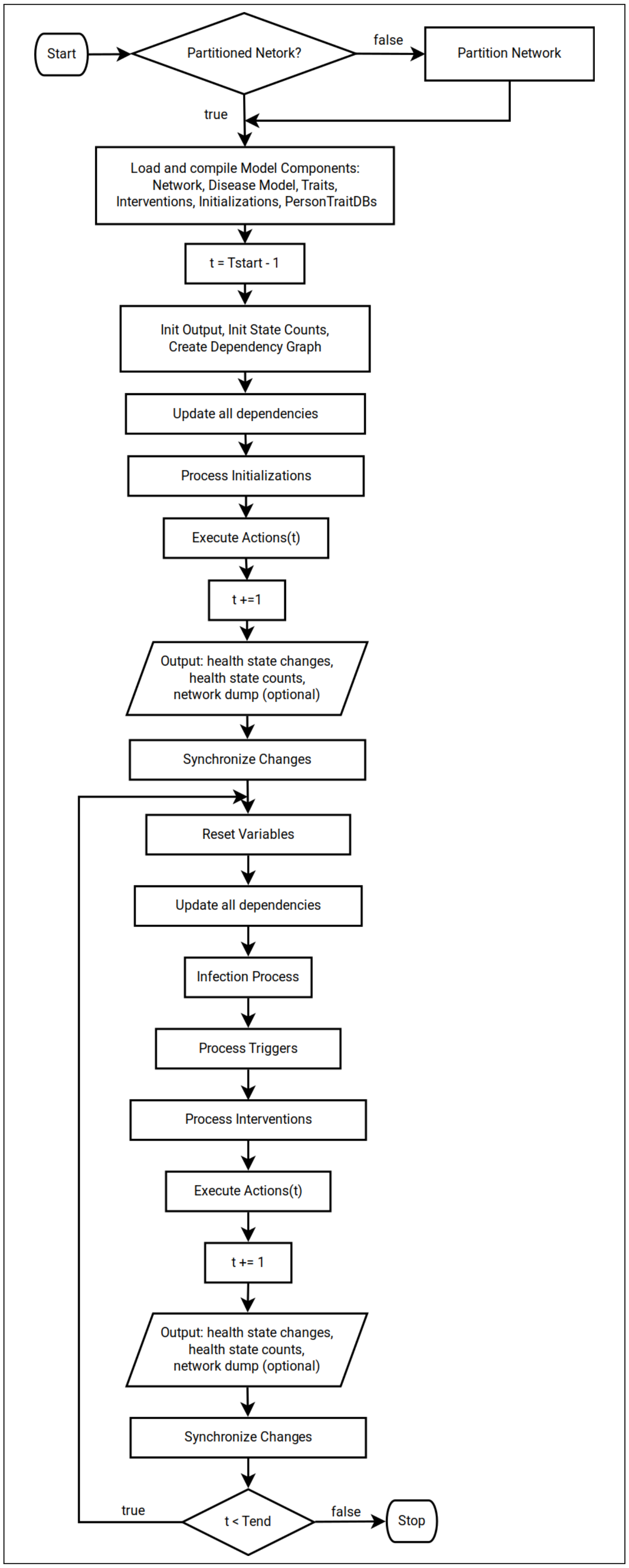

Fig. S2. EpiHiper main algorithm. This flow chart shows the overall implementation of the simulation process, including partitioning and initialization.

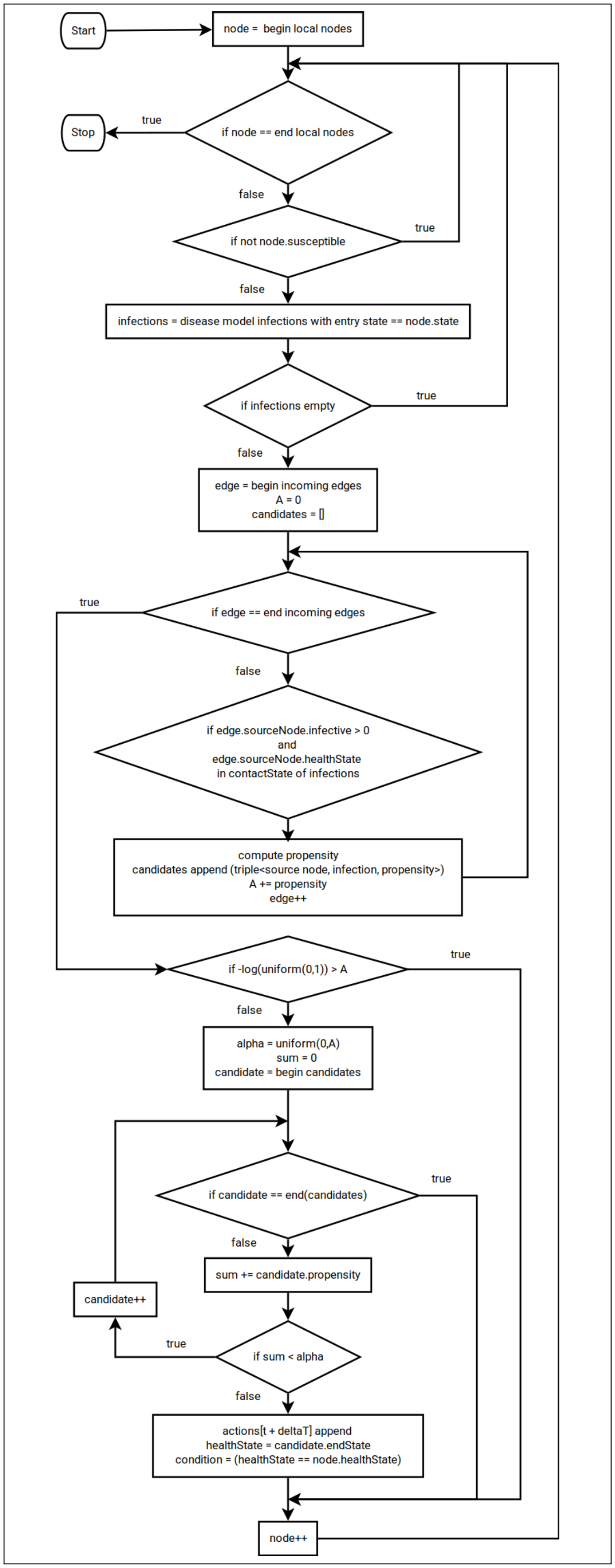

Fig. S3. EpiHiper Transmission processing algorithm.

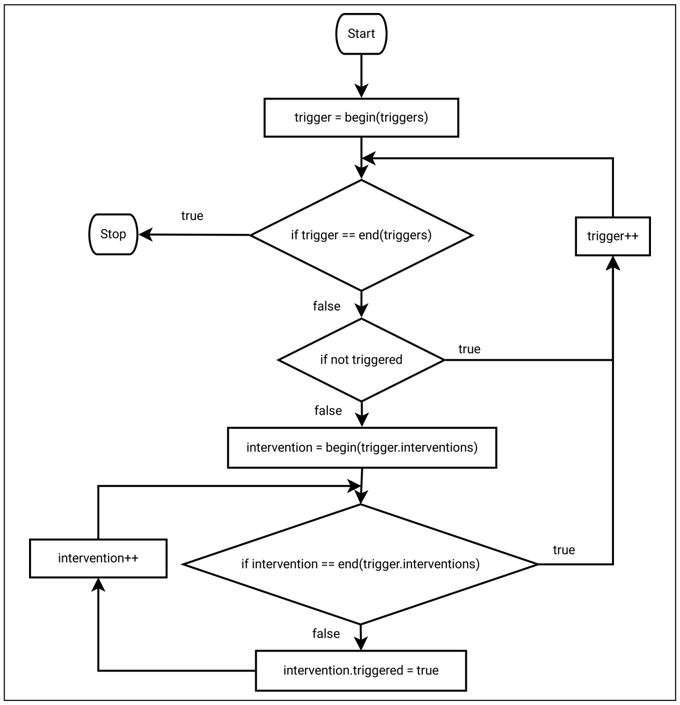

Fig. S4. EpiHiper trigger processing algorithm.

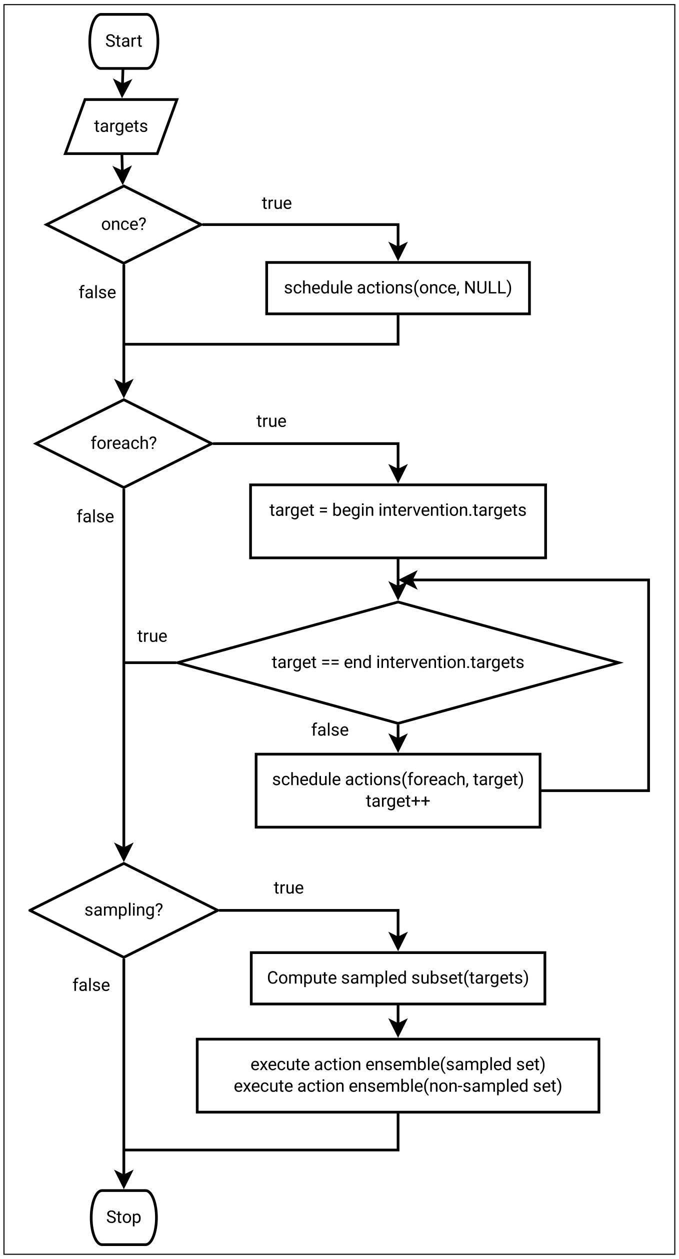

Fig. S6. EpiHiper action ensemble processing algorithm.

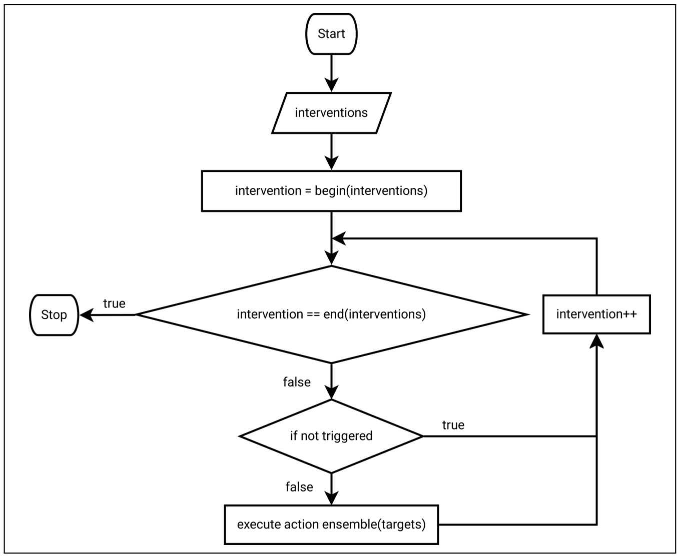

Fig. S5. EpiHiper intervention processing algorithm.

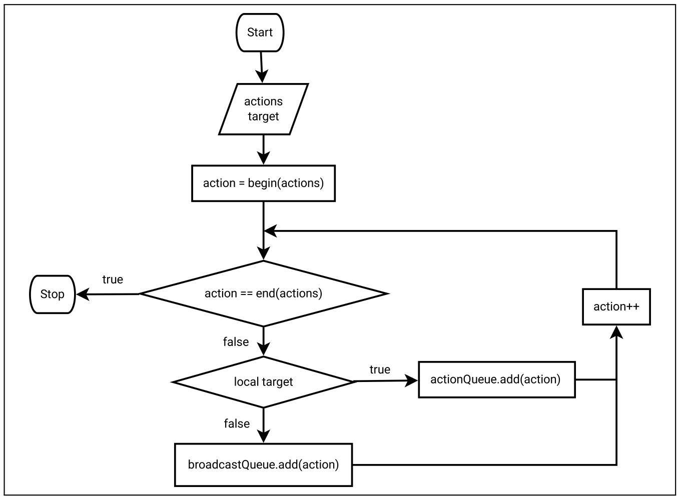

Fig. S7. EpiHiper action scheduling algorithm.

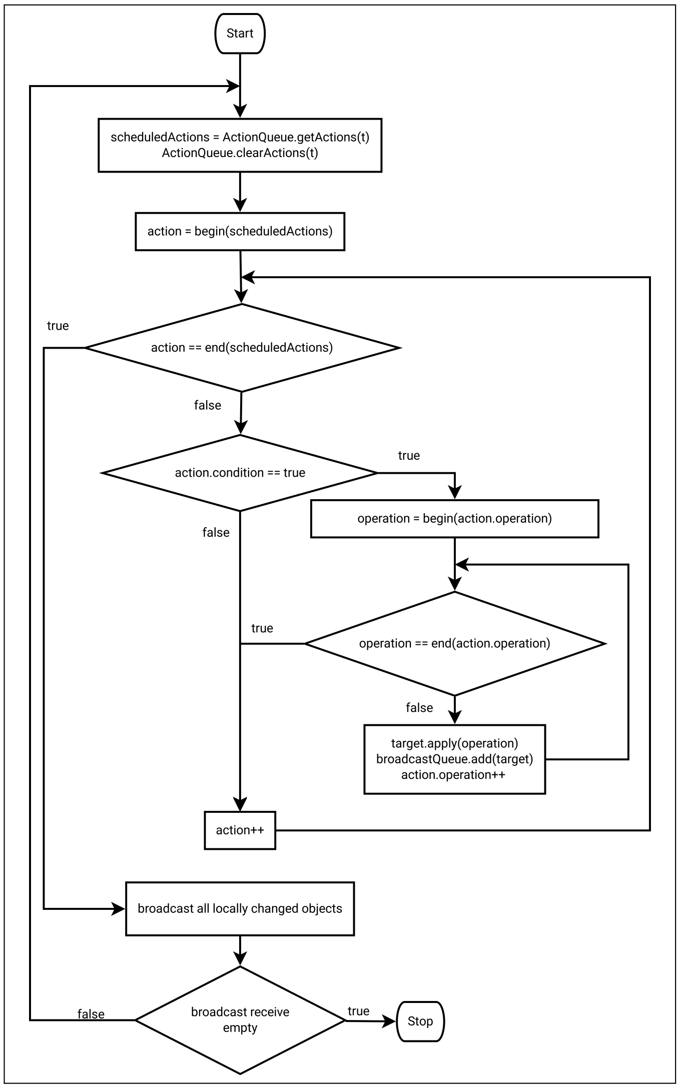

Fig. S8. EpiHiper action execution algorithm.

Table S2. EpiHiper disease models with different complexity beyond SEIR and their computation time.

No.	Disease	Features	Implementation	Complexity			time
				States	Transmissions	Progressions	[s]
I	Influenza	asymptomatic state	add state, transmission, and progression	5	2	4	140 ± 3
II	Ebola			8	3	8	142 ± 4
III	Measles	age stratified	states/transitions for each age group; transmissions across age groups	35	25	20	142 ± 1
IV	COVID v1	severe outcomes	add states and progressions	13	6	16	148 ± 5
V	COVID v2	v1 + age-dependent susceptibility and outcomes	states/transitions for each age group; transmissions across age groups	90	225	100	139 ± 3
VI	COVID v3	v2 + vaccines	vaccinated states with different transitions	105	300	120	146 ± 2
VII	COVID v4	v3 + multivariant	variant-specific infectious states	140	600	185	141 ± 5
VIII	COVID v5	v4 + immune waning/escape	transition from R to S; transmission across variants	170	975	250	140 ± 3

Table S3. EpiHiper interventions and factors influencing their computational complexity.

Intervention	Details	Node Sets	Edge Sets	Set Operations	Traits	Demographics
VHI	Voluntary home isolation	1	2	2	1	2
SC	School closure	0	1	0	1	2
SH, RO, PS	Order, reverse, alternate stay at home order	1	1	1	1	2
TA	Test and isolation of asymptomatic cases	1	5	3	2	2
CTD1	Contact tracing distance 1	4	5	5	3	2
CTD2	Contact tracing distance 2	7	7	8	3	2

D. EpiHiper scaling. Here we provide a detailed description of the scaling studies given in the main paper's Results section. First, computational experiments were conducted for eight disease models (I through VIII) across a set of interventions in the case of Virginia (US). Each experiment was conducted on compute nodes with dual CPUs having 20 cores each and 375 GB total memory across the eight disease replicates, each with its set of interventions, and each instance with 15 replicates. The details of each experiment is given by the following tables:

- Table S2: list of the eight diseases and their complexity elements;
- Table S3: the complete list of interventions used in the experiments along with characteristics impacting complexity;
- Table S4: the specific list of interventions used with experiments I through VIII.

Table S4. The list of interventions used for each of the experiments I through VIII.

Experiment	Interventions
I	VHI, SC, SH
II	VHI, SC, SH, RO, TA
III	VHI, SC, SH, TA
IV	VHI, SC, SH, RO, PS
V	VHI, SC, SH, RO
VI	VHI, SC, SH, RO, CTD1
VII	VHI, SC, SH, RO, CTD1, PS
VIII	VHI, SC, SH, RO, CTD2

The disease models listed in Table S2 are variations of the classical SEIR model with extra features. Their complexity can be represented by the number of states and the number of state transitions. Each model was calibrated so that we have a complete epidemic during the simulation for comparability.

Table S3 shows some common interventions implemented in EpiHiper for various studies. The column "Traits" refers to the usage of custom, time-varying attributes of nodes, whereas the column "Demographics" gives the number of fields accessed in the EpiHiper PostgreSQL person trait database. The demographic information in these experiments is only used during initialization, i.e, does not have any influence on the run time.

Run time as a function of disease and intervention complexity. The results (Figure 6 in the main paper and replicated here as Figure S9) are shown for the eight computational experiments (labeled I through VIII) relating intervention complexity and time complexity for combinations of interventions as listed in Table S3. Here, the computation times are broken out into the main simulation tasks (intervention, transmission, update, synchronization, output, and initialization).

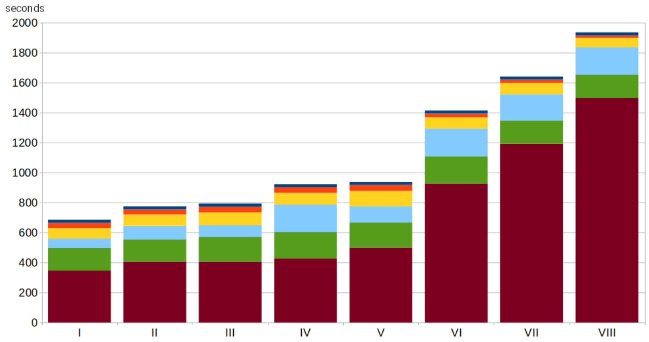

Fig. S9. Impact of intervention complexity on computation time for experiments I–VIII (Table S2) broken out by the main simulation tasks of ■ intervention, ■ transmission, ■ update, ■ synchronization, ■ output, and ■ initialization. The y-axis is measured in seconds.

Run time as a function of network size (nodes/edges). Figure 7 of

the main paper's Results section gives the near linear scaling of run time as a function of the size of the network's node set and edge set in the case of one of our vaccine studies (25) covering all US states.

Memory requirement as a function of network size. In Figure S10, we show the near linear relation between the size of the network's vertex set and the corresponding memory usage for the vaccine study of (25).

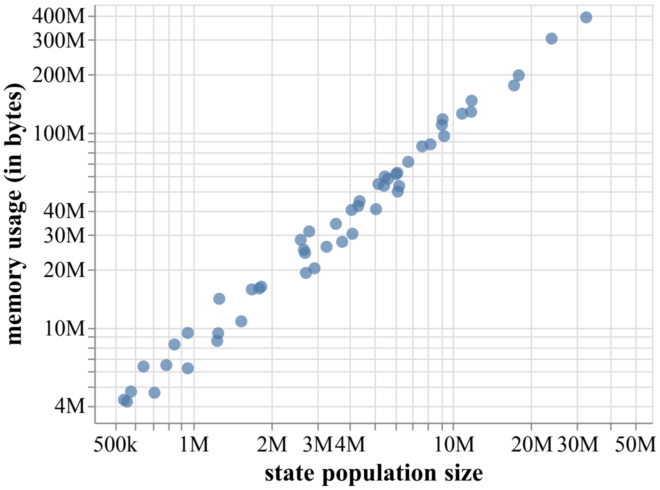

Fig. S10. Scaling of state size vs memory requirements for our vaccine study (25)

Performance and scaling with respect to computational resources. We performed code profiling to determine whether the chosen network partitioning strategy is feasible for different problem sizes. Figure S11 indicates that increasing the number of cores by a factor of 60 (from 2 for small US states (e.g., AK, DC, MT, ND, SD, VT, WY) to 120 for CA; see also Figure 7 of the main paper which provides the number of compute nodes used) only increases the synchronization by a factor of 8 (3.23%–25.2%), indicating that inter-processes communication scales approximately with $\sqrt{N}$ where N is the number of cores supporting the chosen partitioning strategy. The memory and run time requirements for different problem sizes scale linearly with the problem size. In fact, we found that the memory requirements change minimally with varying disease model and intervention complexity. The scaling of the run time, even though always linear, is very much dependent on the complexity of both.

About comparisons of epidemiological simulation tools and their scaling capabilities. When performing a scaling study across tools from a common domain (e.g., epidemiology), it is important to factor in their computational architectures and the restrictions imposed through their respective designs. For reference, EpiHiper was designed to scale to large scenarios and networks. From the requirements, the target networks would require multiple compute nodes and a distributed map of the problem representation into memory across nodes. Moreover, EpiHiper was designed to also take advantage of the cores within a compute node using a threaded model.

Compared to simulation designs that target a single compute node, EpiHiper incurs a communication cost for process synchronization that such applications would generally not experience. For epidemic scenarios and problem sizes that fit on a single node, EpiHiper will indeed show scaling as a function of the number of cores used, but it will likely be somewhat more modest than, for example, (59) and other well-made, single-node, threaded simulation models. On the other hand, for large problem sizes, the memory limitation of a single compute node will bound the problem sizes that tools such as (59) and the other single-node simulation models in Table 2 (main paper) can handle (at least within reasonable time).

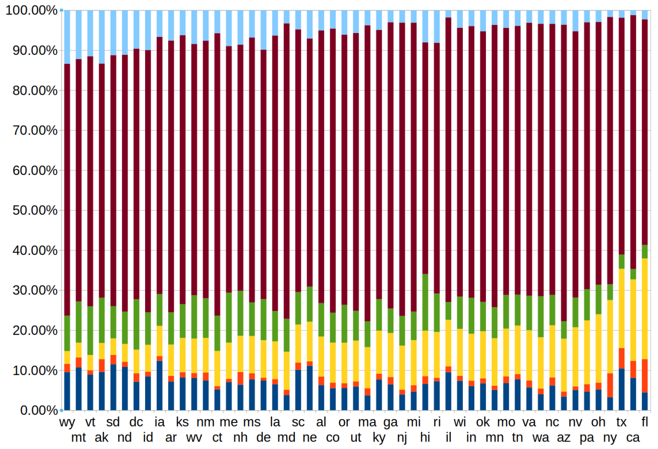

Fig. S11. Distributions showing the breakdown of time across the simulation tasks (i) update, (ii) intervention, (iii) transmission, (iv) synchronization, (v) output, and (vi) initialization) for Round 8 of the Scenario Modeling Hub scenario A (other scenarios lead to similar results). The x-axis specifies the US state (e.g., "wy" for Wyoming) while the y-axis gives the percentage of time used for the respective tasks in the simulation of that state. The states are sorted by the time spent on the synchronization task. As can be seen, a large amount of time is spent on interventions, whereas the disease transmission uses less than $1/4$ of its corresponding intervention time.

For a direct comparison of EpiHiper and other tools, one is thus left with the situation where (a) problem sizes can be addressed by the single-node simulation models in Table 2 (main paper), but for which EpiHiper scaling (as a function of cores) is more modest; and (b) large scenarios and problem sizes well suited for EpiHiper, but that are beyond what those single-node simulation models can handle.

For (53) and other models, the authors have addressed scaling through sampling, which in many cases can provide satisfactory results. While one may therefore argue that some single-node simulation models can scale to larger problem sizes, this is not straightforward and will also require a justification that a down-sampled model version remains valid. Moreover, such an undertaking is something that teams other than the the original one are likely less qualified to be able to carry out, and most certainly for maximal scaling. Based on this, we have therefore chosen to omit detailed scaling comparisons between EpiHiper and the well-established models of Table 2 of the main paper.

We believe, however, that this overview in the Discussion section of the main paper will help readers make informed decisions about which model(s) to adopt and that best fit their needs based on their scenarios and computing resources.

E. A complete EpiHiper example: in-school NPIs. We provide a hypothetical example of EpiHiper-enabled use cases and describe its background and study design, preparation of input files, experiment execution, and some preliminary results from the simulation output. More details can be found in the online documentation[††], with pointers to input and output files that one can use to replicate the experiment or to customize for another study.

E.1. Experiment design. In this example, we study in-school non-pharmaceutical interventions (NPIs) to be implemented in all K-12 schools of a county (Montgomery county, Virginia) when a COVID-19-like disease starts to spread in the county from a few importations. It demonstrates how public health decision makers can use EpiHiper-based simulation experiments to evaluate different intervention policies for situation assessment and/or course-of-action analysis.

Hypothetically, we are interested in the following NPIs:

1. Hybrid learning: schools are open every Monday, Tuesday, and Wednesday for in-person learning, but closed every Thursday and Friday for remote learning.
2. In-school testing:
    - Daily antigen test: test everyone in school (including students and teachers) every in-person day; results come out on the same day with 80% sensitivity.
    - Weekly PCR test: test everyone in school (including students and teachers) every Monday; results come out the next day with 95% sensitivity.
3. Home isolation: students and teachers with positive results are required to stay home for 14 days.

We want to evaluate the effects of these NPIs and to compare the two testing strategies in terms of reducing infections. We consider a base scenario and two intervention scenarios.

- Scenario A: base scenario, no NPIs.
- Scenario B: hybrid learning from day 0; daily antigen test and home isolation from day 30.
- Scenario C: hybrid learning from day 0; weekly PCR test and home isolation from day 30.

E.2. Simulation input files. The person trait database and the contact network file that represent the synthetic population and its network for Montgomery County (VA) is generated as described in Supplementary Material Section F. The network is based on a contact model with $A = 5$, $B = 40$, and $\alpha = 1000$.

This hypothetical example concerns the early stage of a COVID-19-like pandemic. We assume a single virus variant and ignore immunity waning. There is no vaccine yet. Therefore the disease model uses states S (susceptible), E (exposed but not yet infectious), $Ipresymp$ (pre-symptomatic and infectious with a lower infectivity), $Isymp$ (symptomatic with full infectivity), $Iasymp$ (asymptomatic with full infectivity), and R (recovered). The exact parameterization of the states, the transmissions and the transitions can be found in the disease model file (JSON) included with the example.

At the beginning of each simulation, everyone is assumed to be in the S state. The simulation is seeded by 5 infections, randomly selected from all susceptible people, every day for 10 days. This models the start of disease spread in the county from importations.

In the intervention file (JSON), we implement the following: (i) `close_schools` intervention for hybrid learning, which disables all school type edges during the days schools are closed; (ii) `antigen_test` intervention for the daily antigen test, which targets in-school nodes (all individuals studying or working at schools), is triggered every day schools are open, and identifies positive nodes with probability 80%, where a node is positive if it is in one of Ipresymp, Isymp, and Iasymp states; (iii) `PCR_test` intervention for the weekly PCR test, which targets in-school nodes, is triggered every Monday, and identifies positive nodes with probability 95% and one day delay; (iv) `home_isolation` intervention for home isolation, which targets nodes who are tested positive, and remove all edges incident on the target nodes, except home type edges, for 14 days.

All the input files are the same for different scenarios, except for the intervention file. It is customized so that in scenario A no NPIs are ever triggered, in scenario B all NPIs except weekly PCR testing are triggered, and in scenario C all NPIs except daily antigen testing are triggered.

E.3. Results. We run each scenario for 300 days and 30 replicates. Figure S12 is produced from aggregate output data. It shows the cumulative infections as a percentage of the county population over time. The in-school NPIs reduce infections significantly: the overall attack rate (total number of infections normalized by population size) decreases from about 10% in Scenario A to less than 7.5% in Scenarios B and C.

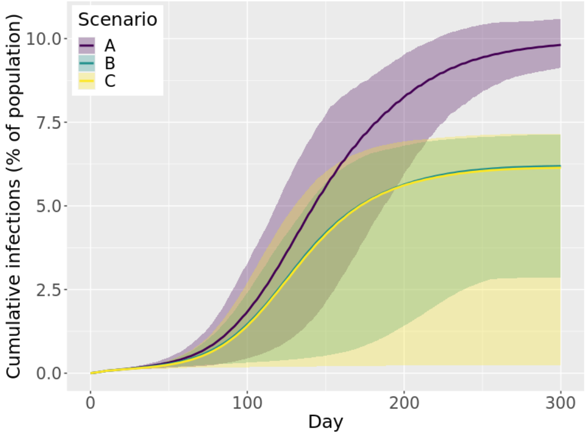

Fig. S12. Cumulative infections over time in different scenarios. NPIs in scenarios B and C significantly reduces infections as compared with scenario A. The curves show the mean values and the ribbons show 90% projection intervals.

Figure S13 is produced from individual level output data, by joining it with the age column in the person trait database. It shows weekly new infections (normalized by age group size) in each of the five age groups (0-4, 5-17, 18-49, 50-64, and 65+), in different scenarios. It seems that in-school NPIs mainly reduce infections in 0-4 and 5-17 age groups, as expected. They only slightly reduce infections in the 18-49 age group, and have little impact on older age groups.

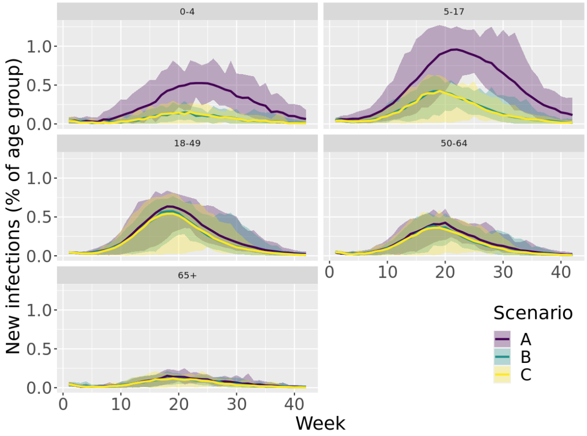

Fig. S13. Weekly new infections by age group in different scenarios. In-school NPIs mainly reduce infections among individuals younger than 18 years old. The curves show the mean values and the ribbons show 90% projection intervals.

This study suggests that in-school NPIs are effective in reducing infections, mainly in the younger population (less than 18 years

[††] https://epihiper.readthedocs.io/en/latest/examples/examples.html

old). To further reduce infections in other age groups, the public health policymakers may consider implementing NPIs in the whole population, including social distancing, testing, and home isolation of positive cases. The effectiveness of these NPIs can be evaluated with an expanded simulation experiment by EpiHiper.

Regarding the smaller impact on the older population, note that in-school measures mainly identify and isolate infected younger people (age < 18 years), and reduce contacts between younger people. For example, 75% of the 0–17 year segment are affected by the hybrid learning measure, while only about 2% of the 18+ segment are affected by the same measure. The reduction of infections in the 0–17 age group means lower infectivity in the population and lower infection risk to the 18+ segment, but this effect is secondary and smaller.

F. Populations and networks. EpiHiper relies on population and network data for the study region R. For this, we use our *digital twins* framework (75, 76) of the actual populations (also referred to as synthetic populations). These are statistically accurate representations of the actual population, and capture (*i*) the people and their household structures, (*ii*) activity schedules for each person to model the activities they conduct (and when) during a typical day or week, (*iii*) the locations where they conduct their activities, and (*iv*) a contact network describing with whom they come in contact during this time; see Figure 8 in the main paper for more details.

More specifically, a digital twin has a *base population*, which is a set P consisting of (digital) people with demographic attributes such as age, gender, race, and work designation (derived from the North American Industry Classification System (NAICS) (77) of the Public Use Microdata Sample (PUMS) (78)). Household structure is central to many scenarios, in particular to epidemiology and the design of interventions. The base population is therefore partitioned into a set H of *households* through the iterative proportional fitting (IPF) (79) process and data contained in the PUMS records. Households are augmented with demographic data, such as household income and number of workers.

Each person in the digital twin population is matched with an *activity sequence* using techniques such as Classification and Regression Trees (CART) and Fitted Values Means (FVM) (80, 81), with data collected mainly from the National Household Travel Survey (NHTS) (74) in the case of digital twins for the United States, but also from sources such as the Multinational Time Use Study (MTUS) and the American Time Use Survey (ATUS) (82, 83). These activity sequences, upon standardization, contain typical activities such as home, work, school, college, shopping, religion, and other, along with the start time and duration of each activity.

The *locations* where people may conduct their activities include residential dwelling units (residence locations) and locations where people conduct their non-home activities, such as work, school, worship, or shopping (activity locations). Locations, which are geographically embedded, are constructed carefully through an ensemble of PostGIS and machine-learning based models fusing NSSAC's extension of the Microsoft Building Data (84) with point-of-interest (POI) data from HERE (85), BuildingFootprintUSA (86), and Scalable Linking and Integration of big POI data (SLIPO) (87). This is augmented with data on school and college locations from the National Center of Education Statistics (NCES) (88), and classification steps based on, for example, land-use polygons and urban/rural classifications (89). Locations, as explained below, represent the places where people interact, and are augmented with North American Industry Classification System (NAICS)-derived designations governing the corresponding designations of people who may work there.

The population construction next performs a *location assignment* that maps people's activities to locations in a manner constrained by rules such as "school activities should happen at schools", "shopping activities must take place in locations supporting retail", and "a student's residence and school location should reside in the same county" (although there are clearly exceptions). Assignment of work locations relies upon the American Community Survey (ACS) commute flow data (90) and LEHD Origin-Destination Employment Statistics (LODES) (91) to accurately capture commuting patterns and long-distance travel, and to match average daily travel distances as reported by the Census (92).

The activity location assignment is succinctly captured by the *people-location network* G_{PL} shown in Figure 8 (middle) of the main paper. From the network G_{PL} we next construct the *social contact network* G_P which has vertex set P. Edges are constructed based on simultaneous visits of people p and p' to the same location ℓ, each such joint visit being a candidate edge. A *contact model* is applied at each location to infer which of the candidate edges become edges $e = (p, p')$ of G_P as illustrated on the right in Figure 8 of the main paper. The contact model used for the networks provided to EpiHiper is an extension of the Erdős-Rényi random graph, and is defined in the following manner: for a location ℓ with a maximal number of simultaneous visits $N = N_\ell$, the per-location contact probability $p_\ell = p_\ell(N)$ is set so that the degree of individuals at the time of the maximal number of simultaneous visits satisfies (in expectation)

$$p_\ell(N-1) = A + (B - A)(1 - e^{-N/\alpha})$$

for non-negative numbers $A < B$ and α, while ensuring that we have $p_\ell \in [0, 1]$. Thus,

$$p_\ell = \min\Big\{1, \Big[A + (B - A)(1 - e^{-N/\alpha})\Big]/[N-1]\Big\} \qquad [7]$$

for $N \geq 2$. The parameter α governs how quickly one (in expectation) reaches the upper bound B. This random graph model is applied to each location ℓ independently, each with contact probability p_ℓ as in Eq. (7). This network modeling approach accomplishes at least two things. First, the temporal component is incorporated: the time intervals of activities/visits of people must intersect for there to be a contact. This resolution, as far as we know, is typically not present in other contact network modeling efforts. Secondly, this approach will, at least partially, avoid the case where nodes get unrealistically large degrees arising from locations with large visit counts (e.g., a school) and the naïve application of the standard Erdős-Rényi model. For calibrating the network parameters of the modified Erdős-Rényi contact model, we used SocioPatterns and POLYMOD data (60–62, 93). Using sampling, sub-graphs induced by particular location types (e.g., schools) were projected from the full contact networks, and the corresponding average degrees were compared to those provided in (60, 62) to ensure reasonable agreement.

The main components of the digital twin are illustrated in Figure 8 (main paper) covering (i) the people, (ii) their residences, retail locations, schools and workplaces, and (iii) their mapping of activities to locations (left side). In the middle of Figure 8, the resulting *people-location network* G_{PL} is illustrated, while on the right, an example contact network G_P is shown. EpiHiper may pre-partition the contact network G_P, and will load the custom person and edge attribute data into its *trait database*.

The population P and the network trait data for $G_P(V_P, E_P)$ are provided to EpiHiper in the form of the *person and edge trait database* (see Supplementary Material Section C). These highly customizable databases allow EpiHiper to efficiently access the digital twin data and to construct, for example, target sets for interventions; see Figure 1 of the main paper. In addition, this design also allows efficient augmentation of person traits by adding, for example, model-based inference of vaccination status and likelihoods of compliance.

G. The Scenario Modeling Hub and EpiHiper. The cases investigated by the SMH focus on studying counterfactual (or what-if) scenarios. As such, predictive accuracy of the models is not the primary goal of the SMH studies. Rather they are interested in providing a range of possibilities should certain scenarios play out. A detailed discussion on this aspect can be found in (94–96). Nevertheless, EpiHiper-based projections were almost always consistent with other projections. When they were not, one could explain why this might not be the case due to inherent assumptions. Please note that each modeling team is allowed to make a number of assumptions and interpret the broad guidelines provided by the scenario design. The basic idea is for policy makers to have a range of outcomes. In this sense, SMH- and Forecast hubs serve different purposes. We have also used EpiHiper-based scenario projections to support the European scenario modeling hub projections https://covid19scenariohub.eu/models.html and, once again, our projections have been consistent with projections provided by other methods.

EpiHiper provides unique insights when supporting scenario projections and supporting policy formation and analysis. For Scenario Modeling Hub rounds, EpiHiper has provided new insights in terms of transmission pathways and spatio-temporal details that other aggregate models in the hub were not able to (see (97, 98)). As discussed, EpiHiper has been used to support other important policy analysis, including: (*a*) spatial and network-based allocation of vaccines (99), (*b*) contact tracing protocols (55), (*c*) economic impact of the pandemic (100, 101). Each of the analyses provided unique insights that would be challenging to obtain based on aggregate models or models that did not have realistic representation of either the network or interventions.

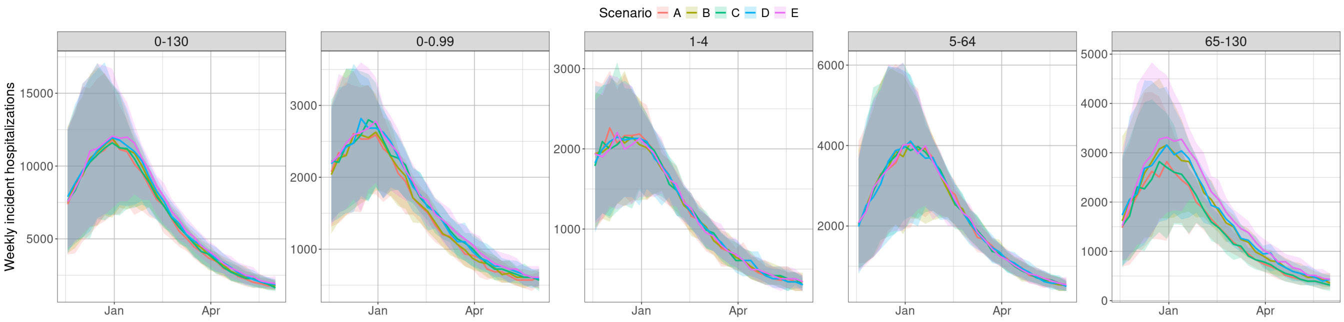

Fig. S14. RSV Scenario Modeling Hub Round 1 projections by the EpiHiper model for hospitalizations under five scenarios (A: optimistic infant and senior protections, B: optimistic infant and pessimistic senior protections, C: pessimistic infant and optimistic senior protections, D: pessimistic infant and senior protections, E: no protections) from November 2023 to June 2024. The curves show the median of the projections, and the ribbons show the 95% projection interval.

H. RSV Scenario Modeling Hub Round 1. At the end of 2023 we used EpiHiper to support Round 1 of the RSV Scenario Modeling Hub. The main goal of this round was to assess the modeling capabilities of the hub for Respiratory Syncytial Virus (RSV) dynamics in the US with limited epidemiological data, and to apply such capabilities to project the impacts of new interventions for the 2023–24 season. Five scenarios were considered: the infant monoclonal axis includes an optimistic protection setting and a pessimistic setting; the senior vaccination axis also includes an optimistic protection setting and a pessimistic setting. The optimistic settings of both axes feature higher coverages and higher efficacies against hospitalization, while the pessimistic settings of both axes feature lower coverages and lower efficacies against hospitalization. An additional counterfactual scenario with neither infant monoclonals nor senior vaccinations was considered to study the impacts of both interventions. The projection target is age specific hospitalizations. Additional details regarding this round can be found at https://github.com/midas-network/rsv-scenario-modeling-hub.

The EpiHiper projections at national level are shown in Figure S14. We found that incident hospitalizations due to RSV infections peak around January 2024 for all age groups. Both interventions have negligible impacts on the age groups 1–4 and 5–64 which are not targeted by the interventions. Even for the infants (below 12 months old), the interventions do not have significant impacts. The senior vaccinations, however, seemed to reduce hospitalizations in the 65+ age group. The results are consistent with the scenario assumptions that monoclonal coverage in the infants is very low even with the optimistic setting, while vaccination coverage is relative high in the senior people, as well as with the modeling assumptions that the infants and the senior are less connected with the rest of the population in the contact network.

I. Additional Related Work. COVID-19 ABM. This computational COVID-19 model by Galvani et al. (102) and used in, for example, (103), is an agent-based simulator with code written in the Julia Language. Its scaling is somewhat limited, focusing on populations of size $\leq 10,000$, and it uses a population structure similar to that of Covasim and OpenABM with network structures-based contact data such as (60). Their source is openly available[‡‡].

FRED. The agent-based model of FRED (31) supports multiple, un-coupled (orthogonal) disease models. As for the models mentioned above, the basic transmission is done per place (location) using a uniform mixing model. The supported models appear to take the form of basic SEIR, SEIS, and SEIRS processes, with opportunities for the user to specify dwell time distributions. As per their documentation, there appears to be support for networks (page 48), but such data does not seem available and is not mentioned in (31). Using their notion of places, the equivalent of a contact network is thus constructed by the epidemic simulator for each time step and for each location. The implementation of FRED as reported in (31) is threaded C++ code using OpenMP. The simplifying assumption of uniform mixing within a location in the contact modeling, which may be adequate in many cases, allows one to omit explicit network representations, in turn permitting the reported scaling to populations of 30 million people. The free version of FRED is openly available[§§]; we remark that the official and commercial versions of FRED are now owned, maintained, and updated by the company Epistemix.

[‡‡] COVID-19 ABM: https://github.com/affans/covid19abm.jl

[§§] FRED: https://github.com/PublicHealthDynamicsLab/FRED

References

1. Halloran ME, et al. (2008) Modeling targeted layered containment of an influenza pandemic in the United States. *Proceedings of the National Academy of Sciences* 105(12):4639–4644.
2. Ferguson N, et al. (2020) Report 9: Impact of non-pharmaceutical interventions (NPIs) to reduce COVID19 mortality and healthcare demand.
3. Marathe MV, Ramakrishnan N (2013) Recent advances in computational epidemiology. *IEEE Intelligent Systems* 28(4):96–101.
4. Lofgren ET, et al. (2014) Opinion: Mathematical models: A key tool for outbreak response. *Proceedings of the National Academy of Sciences* 111(51):18095–18096.
5. Iranzo V, Pérez-González S (2021) Epidemiological models and COVID-19: a comparative view. *History and Philosophy of the Life Sciences* 43(3):104.
6. Borchering RK, et al. (2023) Impact of SARS-CoV-2 vaccination of children ages 5–11 years on COVID-19 disease burden and resilience to new variants in the United States, November 2021–March 2022: A multi-model study. *The Lancet Regional Health-Americas* 17:100398.
7. Chen J, Marathe A, Marathe M (2018) Feedback between behavioral adaptations and disease dynamics. *Scientific reports* 8(1):1–15.
8. Borchering RK, et al. (2021) Modeling of future COVID-19 cases, hospitalizations, and deaths, by vaccination rates and nonpharmaceutical intervention scenarios–United States, April–September 2021. *MMWR Morb Mortal Wkly Rep 2021* 70(19):719–724.
9. Guilbeault D, Becker J, Centola D (2018) Complex contagions: A decade in review. *Complex spreading phenomena in social systems: Influence and contagion in real-world social networks* pp. 3–25.
10. Adiga A, et al. (2020) Mathematical models for COVID-19 pandemic: A comparative analysis. *Journal of the Indian Institute of Science* 100(4):793–807.
11. Eubank S, et al. (2004) Modelling disease outbreaks in realistic urban social networks. *Nature* 429(6988):180–184.
12. Hinch R, et al. (2021) OpenABM-Covid19—an agent-based model for non-pharmaceutical interventions against COVID-19 including contact tracing. *PLOS Computational Biology* 17(7):1–26.
13. Bershteyn A, et al. (2018) Implementation and applications of EMOD, an individual-based multi-disease modeling platform. *Pathogens and Disease* 76(5). fty059.
14. Shattock AJ, et al. (2022) Impact of vaccination and non-pharmaceutical interventions on SARS-CoV-2 dynamics in Switzerland. *Epidemics* 38:100535.
15. Bansal S, Grenfell BT, Meyers LA (2007) When individual behaviour matters: homogeneous and network models in epidemiology. *Journal of the Royal Society Interface* 4(16):879–891.
16. Giabbanelli PJ, et al. (2021) Opportunities and challenges in developing COVID-19 simulation models: Lessons from six funded projects in *2021 Annual Modeling and Simulation Conference (ANNSIM).* pp. 1–12.
17. Pellis L, et al. (2015) Eight challenges for network epidemic models. *Epidemics* 10:58–62.
18. Eubank S, et al. (2004) Modelling disease outbreaks in realistic urban social networks. *Nature* 429(6988):180–184.
19. Bisset KR, Chen J, Feng X, Kumar VA, Marathe MV (2009) Epifast: a fast algorithm for large scale realistic epidemic simulations on distributed memory systems in *Proceedings of the 23rd international conference on Supercomputing.* pp. 430–439.
20. Barrett CL, Bisset KR, Eubank SG, Feng X, Marathe MV (2008) Episimdemics: An efficient algorithm for simulating the spread of infectious disease over large realistic social networks in *SC '08: Proceedings of the 2008 ACM/IEEE Conference on Supercomputing.* pp. 1–12.
21. Bisset KR, et al. (2014) Indemics: An interactive high-performance computing framework for data-intensive epidemic modeling. *ACM Trans. Model. Comput. Simul.* 24(1).
22. Bisset KR, et al. (2012) Simulating the spread of infectious disease over large realistic social networks using Charm++ in *2012 IEEE 26th International Parallel and Distributed Processing Symposium Workshops PhD Forum.* pp. 507–518.
23. Bhatele A, et al. (2017) Massively parallel simulations of spread of infectious diseases over realistic social networks in *2017 17th IEEE/ACM International Symposium on Cluster, Cloud and Grid Computing (CCGRID).* (IEEE), pp. 689–694.
24. Yeom JS, et al. (2014) Overcoming the scalability challenges of epidemic simulations on blue waters in *2014 IEEE 28th International Parallel and Distributed Processing Symposium.* (IEEE), pp. 755–764.
25. Bhattacharya P, et al. (2021) AI-driven agent-based models to study the role of vaccine acceptance in controlling COVID-19 spread in the US in *2021 IEEE International Conference on Big Data (Big Data).* pp. 1566–1574.
26. Bhattacharya P, et al. (2022) Data-driven scalable pipeline using national agent-based models for real-time pandemic response and decision support. *The International Journal of High Performance Computing Applications.* Gordon Bell Award finalist.
27. DrivenData (2022) US-UK Innovation Prize Challenge (https://www.drivendata.org/competitions/98/nist-federated-learning-1/page/525/).
28. DrivenData (2022) PETs Prize Challenge (https://www.drivendata.org/competitions/98/nist-federated-learning-1/).
29. Perumalla KS, Seal SK (2012) Discrete event modeling and massively parallel execution of epidemic outbreak phenomena. *SIMULATION* 88(7):768–783.
30. Skvortsov A, Connell R, Dawson P, Gailis R (2007) Epidemic modelling : Validation of agent-based simulation by using simple mathematical models.
31. Grefenstette JJ, et al. (2013) FRED (a framework for reconstructing epidemic dynamics): an open-source software system for modeling infectious diseases and control strategies using census-based populations. *BMC public health* 13(1):940.
32. Google (2021) Agent based epidemic simulator (https://github.com/google-research/agent-based-epidemic-sim).
33. Kerr CC, et al. (2021) Covasim: an agent-based model of COVID-19 dynamics and interventions. *PLOS Computational Biology* 17(7):e1009149.
34. Fitzpatrick MC, Galvani AP (2021) Optimizing age-specific vaccination. *Science* 371(6532):890–891.
35. Agrawal S, et al. (2020) City-scale agent-based simulators for the study of non-pharmaceutical interventions in the context of the COVID-19 epidemic. *Journal of the Indian Institute of Science* 100(4):809–847.
36. Verity R, et al. (2020) Estimates of the severity of coronavirus disease 2019: a model-based analysis. *The Lancet infectious diseases.*
37. Chinazzi M, et al. (2020) The effect of travel restrictions on the spread of the 2019 novel coronavirus (COVID-19) outbreak. *Science.*
38. Kraemer MU, et al. (2020) The effect of human mobility and control measures on the COVID-19 epidemic in China. *Science.*
39. Peng L, Yang W, Zhang D, Zhuge C, Hong L (2020) Epidemic analysis of COVID-19 in China by dynamical modeling. *arXiv preprint arXiv:2002.06563.*
40. Roosa K, et al. (2020) Real-time forecasts of the COVID-19 epidemic in China from February 5th to February 24th, 2020. *Infectious Disease Modelling* 5:256–263.
41. Grimm V, et al. (2020) The ODD protocol for describing agent-based and other simulation models: A second update to improve clarity, replication, and structural realism. *Journal of Artificial Societies and Social Simulation* 23(2):7.
42. Manninen T, Aćimović J, Havela R, Teppola H, Linne ML (2018) Challenges in reproducibility, replicability, and comparability of computational models and tools for neuronal and glial networks, cells, and subcellular structures. *Frontiers in neuroinformatics* 12:20.
43. Hunter E, Kelleher J (2020) A framework for validating and testing agent-based models: a case study from infectious disease modelling in *34th Annual European Simulation and Modelling Conference.*
44. Hunter E, Kelleher JD (2022) Validating and testing an agent-based model for the spread of COVID-19 in Ireland. *Algorithms* 15(8).
45. Keating SM, et al. (2020) SBML Level 3: an extensible format for the exchange and reuse of biological models. *Molecular Systems Biology* 16(8):e9110.
46. Centola D, Macy M (2007) Complex contagions and the weakness of long ties. *American journal of Sociology* 113(3):702–734.
47. Granovetter M (1978) Threshold models of collective behavior. *American journal of sociology* 83(6):1420–1443.
48. Gillespie D (1976) A general method for numerically simulating the stochastic time evolution of coupled chemical reactions. *J. Comp. Phys.* 22:403–434.
49. Gillespie DT (1977) Exact stochastic simulation of coupled chemical reactions. *J. Phys. Chem.* 81(25):2340–2361.
50. Sze To GN, Chao CYH (2010) Review and comparison between the Wells–Riley and dose-response approaches to risk assessment of infectious respiratory diseases. *Indoor Air* 20(1):2–16.
51. Müller SA, Paltra S, Rehmann J, Nagel K, Conrad TO (2023) Explicit modeling of antibody levels for infectious disease simulations in the context of SARS-CoV-2. *iScience* 26.
52. Horni A, Nagel K, Axhausen K (2016) *Multi-Agent Transport Simulation MATSim.* (Ubiquity Press, London).
53. Müller SA, et al. (2021) Predicting the effects of COVID-19 related interventions in urban settings by combining activity-based modelling, agent-based simulation, and mobile phone data. *PLOS ONE* 16(10):1–32.
54. Li J, Giabbanelli PJ (2021) Identifying synergistic interventions to address COVID-19 using a large scale agent-based model in *Computational Science – ICCS 2021*, eds. Paszynski M, Kranzlmüller D, Krzhizhanovskaya VV, Dongarra JJ, Sloot PM. (Springer International Publishing, Cham), pp. 655–662.
55. Hoops S, et al. (2021) High performance agent-based modeling to study realistic contact tracing protocols in *2021 Winter Simulation Conference (WSC).* (IEEE), pp. 1–12.
56. Lessler J, et al. (2022) COVID-19 scenario modeling hub (https://github.com/midas-network/covid19-scenario-modeling-hub).
57. ScenarioModelingHub (2022) Scenario Modeling Hub Round 8 (https://github.com/midas-network/covid19-scenario-modeling-hub/blob/master/previous-rounds/README_Round8.md).
58. Machi D, et al. (2021) Scalable epidemiological workflows to support COVID-19 planning and response in *2021 IEEE International Parallel and Distributed Processing Symposium (IPDPS).* (IEEE), pp. 639–650.
59. S. F, C. R (2022) Parallelization of large-scale agent-based epidemiological simulations in *Proceedings of the 34th European Modeling & Simulation Symposium (EMSS 2022).*
60. Mossong J, et al. (2008) Social contacts and mixing patterns relevant to the spread of infectious diseases. *PLoS Medicine* 5:1–1.
61. Prem K, Cook AR, Jit M (2017) Projecting social contact matrices in 152 countries using contact surveys and demographic data. *PLOS Computational Biology* 13(9):e1005697.
62. Cattuto C, et al. (2010) Dynamics of person-to-person interactions from distributed RFID sensor networks. *PLOS ONE* 5(7):e11596.
63. IDM (2022) Synthpops (https://docs.idmod.org/projects/synthpops/en/latest/). Last accessed: 22 Jul 2022.
64. Prasanna DR (2009) *Dependency Injection.* (Manning Publications Co.).
65. Zilske M, Nagel K (2017) Software architecture for a transparent and versatile traffic simulation in *Agent Based Modelling of Urban Systems*, eds. Namazi-Rad MR, Padgham L, Perez P, Nagel K, Bazzan A. (Springer International Publishing, Cham), pp. 73–87.
66. Chawla DS (2020) Critiqued coronavirus simulation gets thumbs up from code-checking efforts. *Nature* 582(7812):323–325.
67. Adiga A, et al. (2020) Mathematical models for COVID-19 pandemic: a comparative analysis. *Journal of the Indian Institute of Science* 100(4):793–807.
68. Lucas TC, Pollington TM, Davis EL, Hollingsworth TD (2020) Responsible modelling: unit testing for infectious disease epidemiology. *Epidemics* 33:100425.
69. Ivie P, Thain D (2018) Reproducibility in scientific computing. *ACM Computing Surveys (CSUR)* 51(3):1–36.
70. Open MPI (2024) Open MPI (https://www.open-mpi.org/). Last accessed: 28 July 2024.
71. OpenMP (2024) OpenMP (https://www.openmp.org/). Last accessed: 28 July 2024.
72. Frictionless Data (2024) Frictionless Data (https://frictionlessdata.io/). Last accessed: 24 July 2024.
73. Chen J, Hoops S, Lewis BL, Henning S. Mortveit and SV, Wilson A (2019) Epihiper: Modeling and implementation. NSSAC Technical Report Series: No. 2019–003.

74. U.S. Department of Transportation, Federal Highway Administration (2024) The National Household Travel Survey (NHTS) (https://nhts.ornl.gov.). Last accessed: July 2024.
75. Mortveit HS, et al. (2020) Synthetic populations and interaction networks for the U.S., (NSSAC, University of Virginia), Technical report. NSSAC Technical Report: #2019-025.
76. Adiga A, et al. (2015) Synthetic populations for epidemic modeling. International conference on computational social sciences (ICCSS), June 8-11, Helsinki, Finland, 2015.
77. United States Census (2020) 2017 North American Industry Classification System (NAICS) Manual (https://www.census.gov/library/publications/2017/econ/2017-naics-manual.html). Last accessed: 15 July 2020.
78. U.S. Census (2021) Public Use Microdata Sample (PUMS) (https://www.census.gov/programs-surveys/acs/microdata.html). Last accessed: 24 May 2021.
79. Beckman RJ, Baggerly KA, McKay MD (1996) Creating synthetic baseline populations. *Transportation Research Part A: Policy and Practice* 30(6):415–429.
80. Lum K, Chungbaek Y, Eubank S, Marathe M (2016) A two-stage, fitted values approach to activity matching. *International Journal of Transportation* 4(1):41–56.
81. Breiman L (1984) *Classification and regression trees*, Wadsworth statistics/probability series. (Wadsworth International Group, New York).
82. The University of Oxford (2020) The Multinational Time Use Study (MTUS) (https://www.timeuse.org/mtus). Last accessed: February 2020.
83. United States Department of Labor, Bureau of Labor Statistics (2020) The American Time Use Survey (ATUS) (https://www.bls.gov/tus/). Last accessed: February 2020.
84. Microsoft (2020) U.S. building footprints (https://github.com/Microsoft/USBuildingFootprints).
85. HERE (2023) HERE (http://www.here.com). Accessed April 2023.
86. BuildingFootprintUSA (2020) BuildingFootprintUSA (https://www.buildingfootprintusa.com/). Last accessed 1 April 2020.
87. SLIPO (2021) World-scale OpenStreetMap POIs in CSV (http://www.slipo.eu/?p=1551). Last accessed: June 2021.
88. National Center for Education Statistics (2021) NCES (http://nces.ed.gov). Last accessed: December 2021.
89. U.S. Census (2010) 2010 Census Urban and Rural Classification (https://www.census.gov/programs-surveys/geography/guidance/geo-areas/urban-rural/2010-urban-rural.html). Last accessed: June 2021.
90. United States Census Bureau (2020) 2011-2015 5-year ACS commuting flows (https://www.census.gov/data/tables/2015/demo/metro-micro/commuting-flows-2015.html). Last accessed: April 2020.
91. U.S. Census (2020) Longitudinal Employer-Household Dynamics (https://lehd.ces.census.gov/data/). Last accessed: 26 November 2020.
92. Bureau of Transportation Statistics (2022) Local area transportation characteristics for households data (https://www.bts.gov/latch/latch-data). Last accessed: 31 Jan 2022.
93. SocioPatterns (2023) SocioPatterns (http://www.sociopatterns.org/). Last accessed: 17 Feb 2023.
94. Howerton E, et al. (2024) When do we need multiple infectious disease models? Agreement between projection rank and magnitude in a multi-model setting. *Epidemics* 47:100767.
95. Runge MC, et al. (2024) Scenario design for infectious disease projections: Integrating concepts from decision analysis and experimental design. *Epidemics* p. 100775.
96. Loo SL, et al. (2024) The us COVID-19 and influenza Scenario Modeling Hubs: delivering long-term projections to guide policy. *Epidemics* 46:100738.
97. Chen J, et al. (2024) Role of heterogeneity: National scale data-driven agent-based modeling for the US COVID-19 Scenario Modeling Hub. *Epidemics* p. 100779.
98. Bhattacharya P, et al. (2024) Novel multi-cluster workflow system to support real-time hpc-enabled epidemic science: Investigating the impact of vaccine acceptance on COVID-19 spread. *Journal of Parallel and Distributed Computing* 191:104899.
99. Chen J, et al. (2022) Effective social network-based allocation of COVID-19 vaccines in *Proceedings of the 28th ACM SIGKDD Conference on Knowledge Discovery and Data Mining*. pp. 4675–4683.
100. Chen J, et al. (2021) Epidemiological and economic impact of COVID-19 in the US. *Scientific reports* 11(1):20451.
101. Chen J, et al. (2020) Medical costs of keeping the US economy open during COVID-19. *Scientific reports* 10(1):18422.
102. Shoukat A, et al. (2020) Projecting demand for critical care beds during COVID-19 outbreaks in Canada. *Canadian Medical Association Journal* 19(19):E489–E496.
103. Moghadas SM, et al. (2020) The implications of silent transmission for the control of COVID-19 outbreaks. *Proceedings of the National Academy of Sciences* 117(30):17513–17515.
